# Spatial distribution, source identification, and risk assessment of organochlorines in wild tilapia from Guangxi, South China

**DOI:** 10.1038/s41598-020-72160-x

**Published:** 2020-09-16

**Authors:** Yang Ding, Zhiqiang Wu, Ruijie Zhang, Yaru Kang, Kefu Yu, Yinghui Wang, Xiaobo Zheng, Liangliang Huang, Lichao Zhao

**Affiliations:** 1 School of Marine Sciences, Guangxi University, Nanning, 530004 China; 2 Guangxi Laboratory on the Study of Coral Reefs in the South China Sea, Coral Reef Research Center of China, Guangxi University, Nanning, 530004 China; 3 College of Environmental Science and Engineering, Guilin University of Technology, Guilin, 541004 Guangxi China; 4 College of Resources and Environment, South China Agricultural University, Guangzhou, 510642 China

**Keywords:** Ecology, Environmental sciences, Risk factors

## Abstract

Seventy-five wild tilapia samples from six rivers (ten sites) in Guangxi province were collected and analyzed for 53 organochlorine compounds. DDTs, endosulfan, and PCBs were the most dominant compounds found in this study. Tiandong County (TD) and Guigang City (GG) sites were found to be heavily contaminated with high levels of endosulfan (385–925 ng/g lw) and/or DDTs (20.1–422 ng/g lw). The diagnostic ratios indicated that the residues of DDTs and endosulfan in wild tilapia are associated with historical applications as well as the recent introduction of technical DDTs and endosulfan at some sampling sites. The correlation between total length, body mass, and organochlorines (OCs) was higher than the correlation between age and lipid content. There was no significant correlation between organochlorine pesticides (OCPs) and lipid content. Therefore, for organisms, the feeding intensity (related to length and mass) of fish could better reflect degree of pollution than exposure time (age) of fish. The hazardous ratios for the 50th and 95th percentile data of OCPs and PCBs in fish were both below 1, suggesting that daily exposure to OCPs and PCBs yields a lifetime cancer risk lower than 1 in 10,000.

## Introduction

Organochlorines (OCs), including Polychlorinated biphenyls (PCBs) and organochlorine pesticides (OCPs) have been listed in the first control list of Stockholm Convention (2001) due to their persistence, bioaccumulation, and adverse effects on wildlife and humans. Although OCs has been banned for many years, their residues or metabolites are still detectable in various environmental media in different regions^1–3^. It was reported that approximately 8,000 tons of PCBs were produced as commodities during the 1965s–1980s, and another 10,000 tons were imported during the 1950s–1970s^4^. Most of these chemicals were used as dielectric fluids in electrical appliances and additives in paints. At present, a considerable part of capacitors PCBs have been corroded and leaked due to long storage time, which brings pollution to the surrounding environment^3^.

In the aquatic food chain/web, fish have a higher trophic level and weak ability to eliminate organic pollutants^5^. Therefore, fish are a valuable bio-indicator of pollution in aquatic habitats. The consumption of polluted fish is one of the main sources among many sources of organic pollutants in human body. The intake proportion of POPs in human body by the consumption of fish and fish products can reach 30%, or even higher^6^. Tilapia is one of the aquaculture varieties promoted by United Nations Food and Agriculture Organization (UNFAO), as contributing to solving food shortages and improving human diet. Guangxi is one of the three major tilapia producing areas in China. The total output of tilapia in Guangxi was 308,000 tons in 2016, accounting for 17.3% of the total output of tilapia in China^7^. Twenty-five to thirty percent was exported to developed countries, such as Europe and the United States (US), and the rest was used for local or regional consumption.

For many years, environmental scholars have studied the eastern coastal areas of China, but few have studied the current situation of POPs in the central and western regions. The ecological condition of these rivers has not been well studied. The present study was conducted with the following objectives: (1) determine the residue levels of OCs in wild tilapia collected from the main rivers of Guangxi, South China; (2) obtain detailed information on the spatial distribution and composition pattern to identify possible sources of OCs in the wild tilapia; (3) explore the relationship between OCs congeners and biological parameters, i.e. total length, body mass, age and lipid content; and (4) evaluate the potential health risks of OCs associated with wild fish consumption by residents.

## Materials and methods

The study was approved by the Natural Science Foundation Committee of Guangxi, China. All experiments were performed in accordance with relevant guidelines and regulations set forth in the Coral Reef Research Center of China.

### Study site and sample collection

A total of 75 wild tilapia samples, composed of two species Nile tilapia (*Oreochromis niloticus,* n = 41) and Redbelly tilapia (*Coptodon zillii,* n = 34) (see Supplementary Fig. S1 online), were collected from ten sampling sites located in the main rivers in the southern Guangxi during April and October 2018 (see Supplementary Text S1 online). Among these sampling sites, one site (Wuzhou City (WZ)) was located on the main stem of the Xijiang River and nine sites were located on main tributaries (two on Youjiang River (Tiandong County (TD) and Long’an County (LA)), two on Zuojiang River (Chongzuo City (CZ) and Fusui City (FS)), two on Yujiang River (Nanning City (NN) and Guigang City (GG)), one on Qianjiang River (Wuxuan County (WX)), and two on Xunjiang River (Pingnan City (PN) and Tengxian County (TX)) (see Supplementary Fig. S2 online).

We collected three to ten tilapia replicate samples at each sampling point via trawling nets, and the biological parameters (including length and mass) of each fish were measured after collection (see Supplementary Table S1 online). All organism samples were packaged separately in aluminum foil marked with identifying information, transported to the laboratory in refrigerator, and stored at -20 °C.

### Sample preparation and purification

The purchasing sources and handling procedures for all the chemicals and materials used in this work are summarized in Supplementary Text S2 and Table S2. The tilapia samples were defrosted and muscle tissue (without skin) was obtained. The extraction of the muscle samples, the separation of the analytes from the lipids, and the fractionation of the purified extract were carried out following the method described by Ding et al.^8^. Briefly, the muscle samples were lyophilized and ground into a fine powder, spiked with 20 ng surrogate standards (2.4.5.6-tetrachloro-m-xylene (TCMX), PCB30, PCB204), and then Soxhlet-extracted with a mixture of n-hexane and acetone (1:1, v: v) for 48 h. The extraction solution was concentrated to 4 ml with a rotary evaporator. A portion of the extract (1 ml) was used to determine the lipid content (see Supplementary Table S1 online). The remaining extraction was concentrated to 0.5 mL, and was purified using a gel permeation chromatography column (packed with Bio-beads SX-3) and a multilayer silica column to remove lipids^8^. The extract was concentrated to 50 μL under nitrogen stream and ^13^C_12_-PCB138 was added as an internal standard before GC–MS analysis.

### Instrumental analysis

Fifty-three OC congeners were selected as target analytes. These included dichlorodiphenyltrichloroethanes (∑DDTs): *o,p', p,p'*-DDD, -DDE, -DDT; hexachlorocyclohexanes (∑HCHs): α, β, γ, δ-HCH; ∑Drins (aldrin, endrin, dieldrin, endrin aldehyde, endrin ketone);∑CHLs: (trans-chlordane (TC), cis-chlordane (CC), heptachlor, heptachlor endo-epoxide, heptachlor exo-epoxide), endosulfan: (α-, β-endosulfan, and endosulfan sulfate); p,p'-Methoxychlor (MXC); hexachlorobenzene chlordanes (HCB); and PCBs (numbers 8, 18, 28, 44, 52, 66, 77, 81, 101, 105, 114, 118, 123, 126, 128, 138, 153, 156, 157,167, 169, 170, 180, 187, 189, 195, 206 and 209). The basic physicochemical properties of these OCs are summarized in Supplementary Table S2.

The target OCs were quantitatively analyzed using an Agilent 7890B gas chromatograph-7000C tandem mass spectrometry (GC–MS/MS) with electron impact ionization (EI). An HP-5MS GC column (30 m × 0.25 mm × 0.25 μm, Agilent Technologies Inc.) was used to separate the analytes. The GC oven temperature was programmed as follows: 80 °C (5 min) → 20 °C/min → 160 °C (0 min) → 4 °C/min → 240 °C (0 min) → 10 °C/min → 295 °C (2 min). Helium was used as the carrier gas. One μL of sample was injected at constant port temperature of 250 °C. Multi-reaction monitoring (MRM) model was used for mass spectrometry.

### Quality Assurance/Quality Control

A standard solution (2 ng/mL for each OC compounds) with a fixed concentration was injected every day to monitor the sensitivity of the instrument (Text S3). Procedural blanks (n = 8), spiked blanks (n = 8), and spiked matrices (n = 8) were set to ensure method quality control. No targeted OC congeners were detected in the procedural blank. The spiked compounds included 24 OCP congeners and 28 PCB congeners. The mean recoveries of the surrogates in all samples were 72.3 ± 8.4%, 87.6 ± 20.0%, and 92.4 ± 17.8% for TCMX, PCB30, and PCB204, respectively. The final reported concentrations were not adjusted by surrogate recoveries. Recovery of OC standards was 68.6–96.2% in the spiked blanks and 73.8–105.6% in the matrix-spiked samples, with a relative standard deviation of < 17%. The limit of detection (LOD) and limit of quantification (LOQ) for the OCs were defined as the concentrations corresponding to the signal-to-noise (S/N) ratio of 3 and 10, respectively (see Supplementary Table S3 online). The LODs and LOQs for the tilapia samples were 0.006–0.087 ng/g lw (lipid weight) and 0.020–0.289 ng/g lw.

### Statistical Analysis

Concentrations of OCs were expressed in ng/g lw. Values below the limit of detection were noted as zero. Statistical analysis was performed with SPSS 17.0 (SPSS Inc., Illinois, USA). A pearson correlation was used to evaluate the correlation between the sub-groups. When the data were normally distributed, an independent sample *t*-test was applied to various variables to examine the statistical significance of the differences between sub-groups. Otherwise, a non-parametric test was used. A *p*-value of < 0.05 was considered significant while *p* < 0.01 was considered highly significant. A Principal Component Analysis (PCA) was used to investigate the relationship between the contents of the OC compounds and biological parameters. For samples with concentrations below LOD, zero was used in the calculations. For samples with concentrations below LOQ, a value of 1/2 LOQ was used for data analysis.

## Results and discussion

### Occurrence of the target OCs

The following individual OCs were found in fish samples with a detection frequency lower than 50%: i.e. p,p'-methoxychlor, heptachlor, heptachlor exo-epoxide, CB-66, 77, 81, 105, 114, 118, 123, 126, 128, 138, 153, 156, 157,167, 169, 170, and 180. These OCs are not discussed later. The concentrations of seven OC compounds in the muscle samples of 41 Nile tilapia and 34 Redbelly tilapia are shown in Supplementary Table S4. Since there were no significant interspecies differences between Nile tilapia and Redbelly tilapia (t-test, *p* = 0.16), the results of OCs analysis will be reported by tilapia genus in this study.

Median concentrations of OCPs and PCBs in tilapia samples from the main rivers system in the southern Guangxi are summarized in Table 1. PCBs and OCPs were detected in the muscle of all tilapia samples. DDTs were the predominant contaminant with a median concentration of 15.2 ng/g lw, and endosulfan was the second most common contaminant with a median concentration of 12.2 ng/g lw. PCBs, Drins, HCB, and HCHs concentrations in the fish were relatively low with median concentrations between 1.37 and 9.11 ng/g lw. The concentrations of the various OC compounds measured in the tilapia samples in this study were lower than those in tilapia collected from Guangdong province, China^9^, Africa^10–12^, Europe, and America^13–15^ (see Supplementary Table S5 online). This study showed that the main rivers in the southern Guangxi have low levels of OCs pollution, and the fish muscle contamination might be related to the low levels of pollution in the water and sediment. According to data from the National Bureau of statistics of China, the gross output value of industry and agriculture in Guangxi has been lower than that of other provinces or regions in China in the past few decades^16^. Therefore, the low levels of OCs pollution found in this study area are mainly the result of lower pollution input. In addition, most of the study area is located in the tropics, which have a relatively high perennial temperature. A warm climate is very conducive to enhance the metabolism rate of OCs by organisms^17^. The metabolism of organic pollutants by organisms occurs under the catalysis of a series of enzymes^18,19^. Factors affecting the enzymatic reaction, such as enzyme concentration and temperature, will affect the metabolism of OCs in organisms. Temperature also affects the air–water partitioning, which influences the volatilization of chemical pollutants from water^20^. Thus, dissolved chemical concentrations tend to be higher in cooler than in warmer waters^21^. In alignment with this supposition, Sobek et al. (2010) reported a largely reduced difference in bioaccumulation factor of PCBs between the Arctic and the temperate food webs, after adjustment for temperature effect^22^.

**Table 1:** Organochlorine concentration [median (range), ng/g lw] in the wild tilapia from the main rivers in Guangxi, South China.

Sites	N	HCHs^a^	DDTs^b^	CHLs^c^	Endosulfan^d^	Drins^e^	HCB	OCPs	PCBs^f^
TD	10	0.72 (0.47–0.98)	7.59 (4.50–10.2)	5.82 (1.76–9.11)	651 (385–925)	4.81 (3.71–8.21)	1.34 (0.86–1.84)	**672 (400–949)**	**8.38 (7.04–12.2)**
LA	3	1.08 (1.05–1.41)	18.8 (17.5–22.5)	3.11 (2.52–10.8)	13.34 (8.58–22.3)	10.2 (7.89–13.4)	2.36 (2.05–2.47)	**55.0 (47.7–58.7)**	**11.4 (10.5–11.9)**
CZ	8	1.26 (0.79–1.43)	13.2 (8.95–38.3)	1.39 (1.16–2.81)	10.8 (6.61–15.6)	7.15 (5.69–10.7)	3.03 (2.16–3.86)	**38.2 (28.9–66.9)**	**9.38 (7.35–11.4)**
FS	7	1.35 (0.82–2.26)	11.0 (5.98–16.0)	2.43 (1.91–3.83)	9.84 (6.38–36.4)	8.58 (5.64–12.8)	2.95 (2.12–8.21)	**38.0 (34.2–62.5)**	**9.81 (8.34–29.3)**
NN	9	0.88 (0.48–1.77)	11.5 (4.57–26.4)	1.52 (0.90–6.34)	9.38 (6.88–15.2)	6.45 (2.83–20.0)	3.18 (1.71–5.35)	**34.9 (27.1–61.6)**	**9.11 (6.56–17.6)**
GG	9	2.93 (1.73–3.50)	155 (20.1–422)	1.10 (0.59–2.15)	14.9 (7.76–17.6)	5.24 (1.95–10.5)	3.36 (2.67–4.65)	**183 (46.0–452)**	**8.41 (3.36–11.9)**
WX	5	1.17 (0.81–1.40)	12.0 (8.43–15.4)	2.46 (2.39–3.14)	6.48 (5.20–10.7)	10.8 (4.50–12.3)	1.60 (1.38–3.95)	**34.7 (25.6–43.7)**	**9.46 (6.90–15.2)**
PN	10	1.58 (1.30–2.34)	18.3 (5.06–68.9)	0.89 (0.43–1.87)	8.69 (6.83–13.4)	4.43 (2.11–13.0)	2.94 (2.24–5.65)	**36.0 (23.3–93.6)**	**6.67 (3.44–13.5)**
TX	9	1.95 (0.94–3.49)	50.3 (31.4–88.9)	1.81 (1.05–2.75)	3.66 (2.55–10.6)	6.35 (4.25–15.1)	3.16 (1.63–6.00)	**73.8 (46.3–114)**	**17.1 (7.59–19.9)**
WZ	5	4.99 (2.11–5.61)	17.6 (6.65–25.9)	2.02 (1.24–3.17)	10.6 (7.34–22.0)	5.47 (3.18–11.0)	4.95 (0.71–5.56)	**52.5 (30.9–53.6)**	**7.23 (2.65–11.0)**
Total	75	1.37 (0.47–5.61)	15.2 (4.50–422)	1.81 (0.43–10.8)	10.2 (2.25–925)	6.34 (1.95–20.0)	2.94 (0.71–8.21)	**52.5 (23.3–949)**	**9.11 (2.65–29.3)**

^a^Sum of α-HCH, β-HCH, γ-HCH and δ-HCH; ^b^Sum of o,p', p,p'-DDD, -DDE, -DDT; ^c^Sum of TC, CC, heptachlor endo-epoxide; ^d^Sum ofα-, β-endosulfan, and endosulfan sulfate; ^e^Sum of Aldrin, Endrin, Dieldrin, Endrin aldehyde and Endrin ketone; ^f^Sum of CB-8, 18, 28, 44, 52, 101, 189, 195, 206, 209.

### Distribution characteristics

#### Spatial distribution of OCs

**Figure 1:**
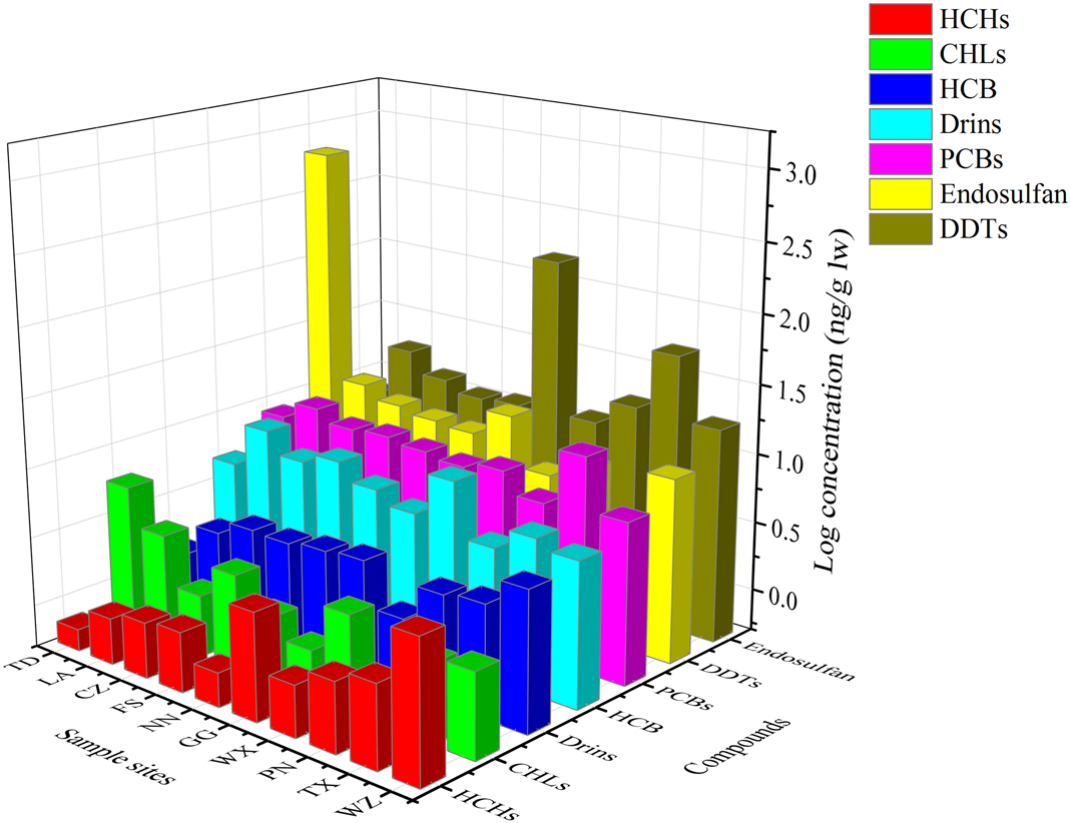
Spatial variations of log-transformed concentrations of OC compounds (ng/g lw) residues in wild tilapia from the main rivers in Guangxi, South China. TD: Tiandong County; LA: Longan County; CZ: Chongzuo City; FS: Fusui City; NN: Nanning City; GG: Guigang City; WX: Wuxuan County; PN: Pingnan City; TX: Tengxian County; WZ: Wuzhou City.

The spatial distributions of seven OC compounds are presented in Fig. 1. The spatial distribution did not show a gradient in selected OCs concentrations. The spatial distribution of OCPs was under the double influence of a global distillation effect and the usage of OCPs^23^. Human activities can affect the distribution of OCPs in hilly areas^24^. However, there was no significant correlation between elevation and the residues of OCPs in this study (non-parametric test, *p* > 0.05) (Fig. S2). Therefore, the distribution pattern of OCs in this study was hardly affected by global distillation. High levels of OCPs were found in TD and GG, where endosulfan or DDTs were the predominant contributors. Endosulfan is a cyclodiene pesticide extensively used throughout the world to control a wide variety of insects and mites^23^. Endosulfan levels were remarkably higher (10–411 times) in tilapia samples from TD than in samples from other sites. This observation was consistent with the fact that the local fruit and vegetable farming industry is the primary income source in the TD^25^. Therefore, we believe that the high levels of endosulfan in this study could be attributed to local pesticide practices specific to pest control needs over a short period^26^. Similarly, the higher levels of DDTs observed in GG also might be related to local short-term agricultural activities.

PCBs are ubiquitous in tilapia samples from the study area, with a detection rate of 100%. In contrast to the OCP compounds, the overall trend of the PCBs was fairly homogenous. A relatively high median PCB concentration was detected in tilapia samples from TX, while slightly lower concentrations were detected from PN. There were no significant differences among different sampling locations (*t*-test, *p* > 0.05). The minor differences could be explained by the migration and spread of PCBs in the environment. The limited historical use of PCBs in the present study area is another important factor contributing to this phenomenon^25^.

#### Spatial differences in pollutant metabolites

**Figure 2:**
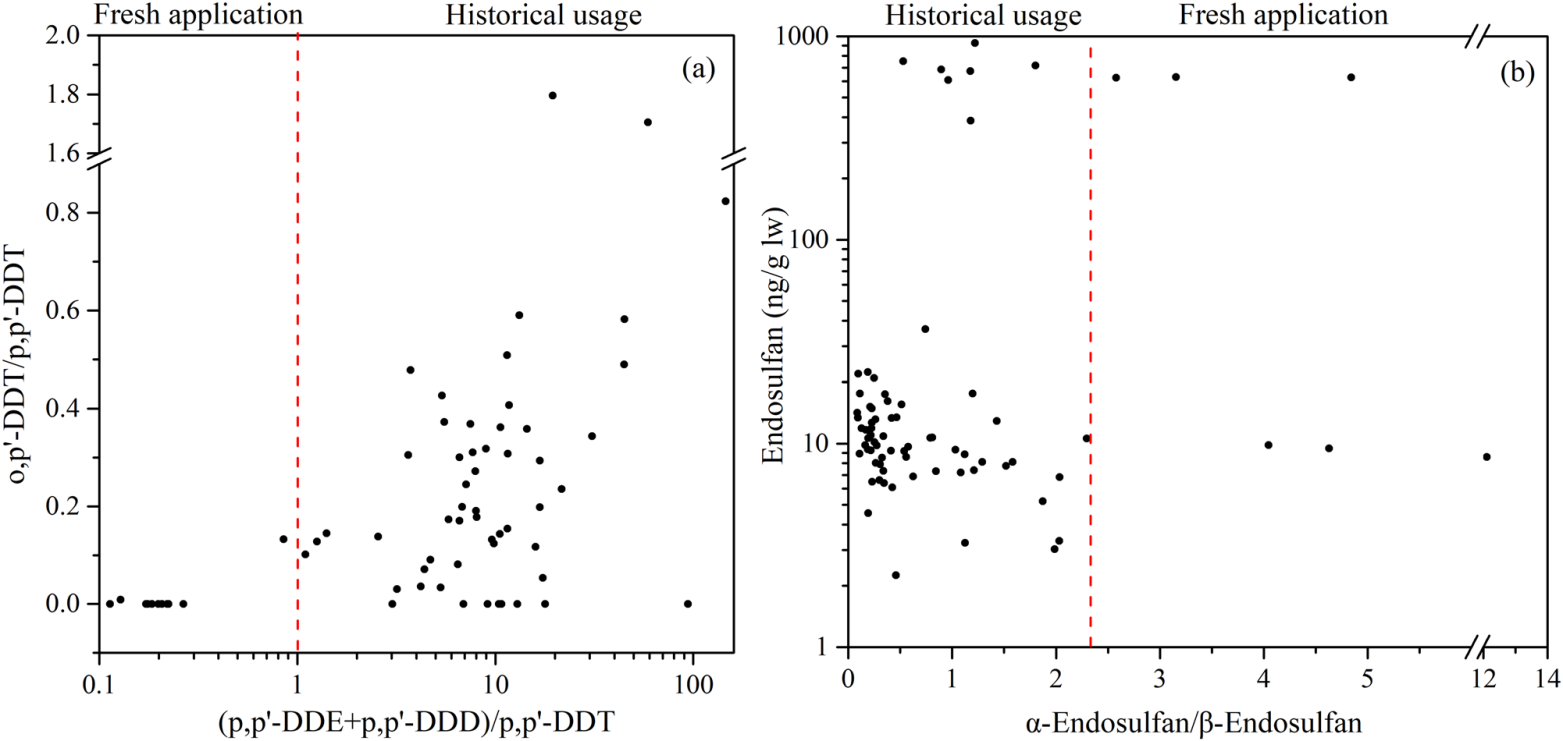
Scatter plots of molecular indices to identify DDTs (a) and endosulfan (b) contamination sources.

The ratio of parent compounds to their metabolites can provide useful information for the diagnosis of their sources^23,24,27^. The scatter plots for isomeric ratios of selected OCPs are shown in Fig. 2.

The ratios between p,p’-DDT, p,p’-DDE and p,p’-DDD have been regarded as an indication of increasing or decreasing inputs to the environment. A ratio of (p,p’-DDE + p,p’-DDD)/p,p’-DDT < 1.0 indicates a fresh application and a ratio of more than 1.0 indicates historical DDTs^23,28^. In the present study, the ratios of (p,p’-DDE + p,p’-DDD)/p,p’-DDT were all higher than 1.0, except in TD. Furthermore, p,p’-DDT was not detected in fish samples from NN, TX and WZ. These two facts imply that there is no recent introduction of technical DDT to the river in these regions (Fig. 2a and Table S6). The ratio of (p,p’-DDE + p,p’-DDD)/p,p’-DDT for the TD site indicated the fresh application of technical DDTs in this area. The major source of DDT pollution in China is through the application of technical DDTs and Dicofol in agriculture^3,24^. Technical DDT contains higher than 85% of p,p-DDT, and less than 15% of o,p-DDT^3^. Therefore, the ratio of o,p’-DDT/p,p’-DDT also can be used to determine whether DDT pollution is caused by technical DDT or Dicofol^29^. Generally, values of o,p’-DDT/p,p’-DDT in the range of 1.3 to 9.3 or higher is indicative of a Dicofol source, while a range of 0.2–0.3 is indicative of a technical DDT source^23^. Our study shows o,p’-DDT/p,p’-DDT ratios predominantly ranging between 0 and 1.80. Higher o,p’-DDT/p,p’-DDT ratios (> 1.3) were found in two fish samples from FS (1.80) and CZ (1.71) districts, which indicates that Dicofol may be the main contributor to DDTs in these areas. In summary, the DDT residues in wild tilapia from rivers of the southern Guangxi originated mainly from the historical application of Dicofol and technical DDTs, whereas recent application of technical DDTs are indicated in TD.

Technical endosulfan includes two active ingredients: α-endosulfan (70%) and β-endosulfan (30%)^23^. Because α-endosulfan decomposes more easily than β-endosulfan, a α-/β-endosulfan ratio of < 2.33 can be used to distinguish between historical use and recent use. In fish samples in which α- and β-isomer were co-detected, the isomer ratios ranged from 0 to 2.1 (Fig. 2b). The higher values of the α-/β-endosulfan ratio (> 2.33) present in the tilapia samples from TD and FS, indicate continual use of endosulfan in these areas. In the other sites, those ratios are all below 2.33, indicating there was no recent application of technical endosulfan in that area. It is noteworthy that one sample (from TX site) contained β-endosulfan at a level below the limit of detection, but had appreciable levels of α-endosulfan, which may have been transported in from other areas. Because the Henry's law constant for α-endosulfan is higher than the constant for β-endosulfan, there is a greater tendency for α-endosulfan to evaporate from the surface medium to air^23,30^.

**Figure 3:**
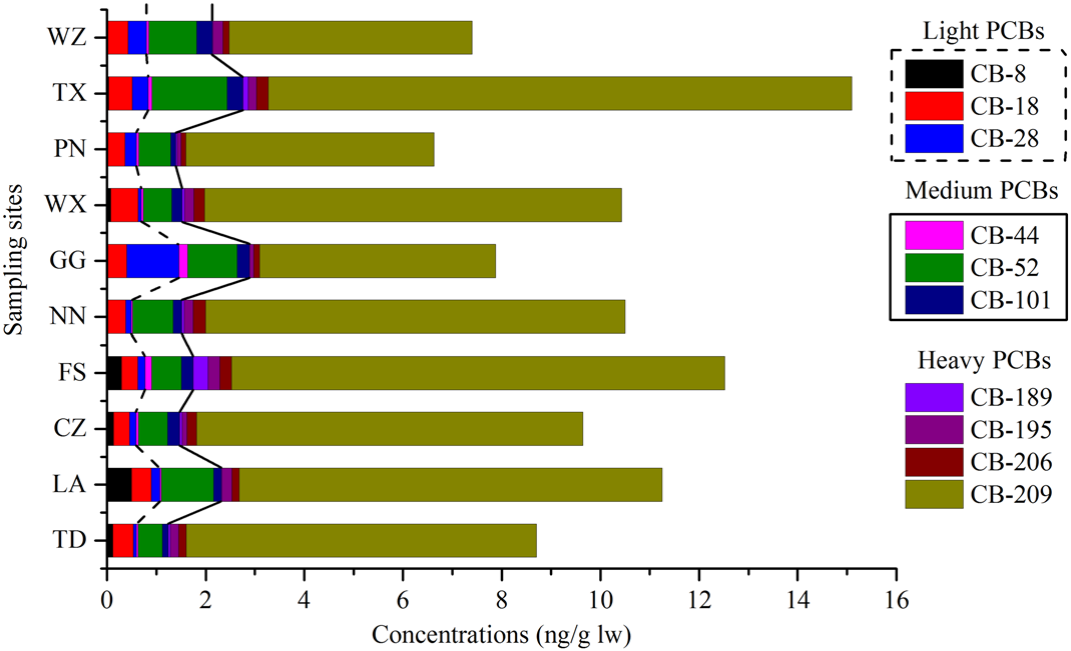
Composition profiles of PCB congeners in the main rivers from Guangxi, South China.

The concentrations of ten PCB congeners in the present study area are illustrated in Fig. 3. Using degree of chlorination, these congeners can be divided into light PCBs (2–3 chlorines), medium PCBs (4–6 chlorines), and heavy PCBs (7–10 chlorines). The PCB sources of the 75 fish samples can be classified into the same categories since the PCBs in all sampling sites generally exhibited the following order: heavy PCBs (63.3–86.1% of ∑_10_PCBs) > medium PCBs (9.72–18.2% of ∑_10_PCBs) > light PCBs (4.66–18.3% of ∑_10_PCBs). The higher residual content of heavy PCBs may be related to historical production and use, or relate to their stably chemical structure^31^. Tri-CBs and penta-CBs were the major PCB products manufactured in China from 1965 until they were banned in 1974^31,32^. The proportion of these compounds in PCBs was only 2.86–24.7% in this study. On the other hand, light PCBs have higher volatility and a lower octanol–water distribution coefficient than heavy PCBs^33^. Once absorbed into the organisms, light PCBs are usually more rapidly metabolized than the more highly chlorinated congeners^34^. Our results also indicated that the proportion of deca-CBs in heavy PCBs and PCBs was 78.3–98.1% and 51.8–88.6%, respectively. And the sampling sites with high deca-CBs ratio were distributed in the main agricultural farming areas (middle and upper reaches of rivers). And China banned the production of deca-CBs as early as 1974^35^. Therefore, we believe that historical heritage was the main source of deca-CBs in the study area.

### Correlation among biological parameters and OC compounds

**Figure 4:**
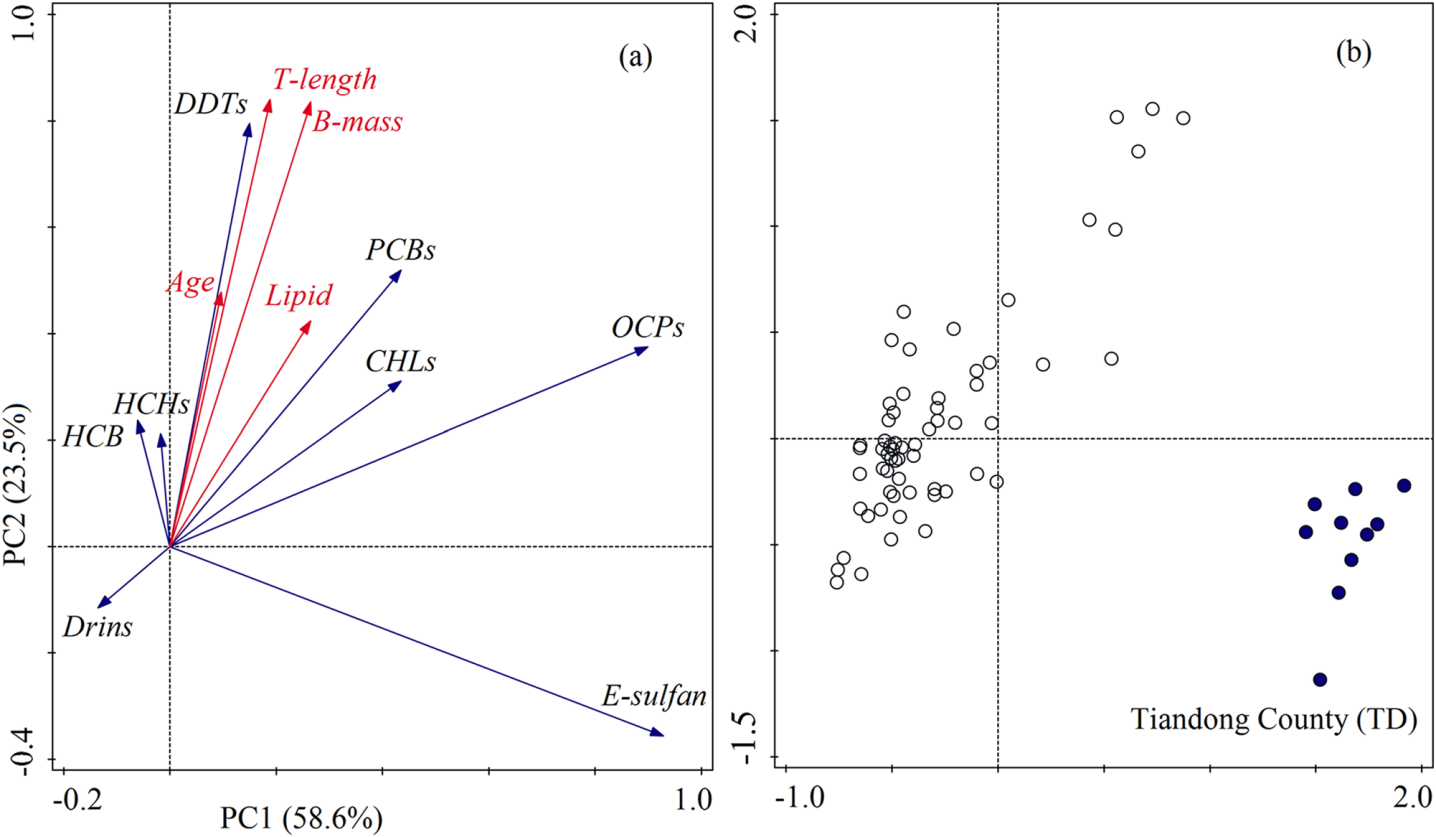
P Loading plot (**a**) of PCA for biological parameters and OC compounds (ng/g dw) (n = 75); and score plot (**b**) of PCA based on the concentrations of OCs in the tilapia samples. T- length: total length; B-mass: body mass; E-sulfan: endosulfan.

We studied the effects of biological parameters (including total length, body mass, age, and lipid content) on the bioaccumulation of individual contaminants in tissue samples of wild tilapia (based on dry weight). The loading and scores plot of PCA based on the concentrations of OCs in the tilapia muscle samples are displayed in Fig. 4. The PC1 explained 58.6% of the total variance and PC2 accounted for 23.5% of the variance. Table S7 lists the correlation coefficients between OC compounds and biological parameters, while the correlation coefficients between OC congeners and biological parameters are listed in Table S8. A significant relationship between growth parameters (i.e. total length, age, and body mass) was found in the tilapia samples, but only age and lipid content were significantly correlated (*p* < 0.01) (Fig. 4 a). The level of OCs in TD was higher than in other sampling sites and was significantly correlated with endosulfan concentration (*p* < 0.05) (Fig. 4 b). PCA analysis showed that DDTs, CHLs, OCPs and PCBs were significantly correlated with the biological parameters, suggesting that these compounds were continuously accumulated during the growth of the tilapia. Surprisingly, there was no significant correlation between OCPs and lipid content, which is different from previous studies^35–37^. Drins and endosulfan were negatively correlated with growth parameters and lipid content. There may be two main reasons for this result. First, endosulfan is likely to be degraded in tilapia because the bioaccumulation capacity of endosulfan is poor (log *K*_*ow*_ = 3.66) when compared to other compounds^38^. This also may be the main reason for the poor correlation between OCPs and biological parameters. Secondly, Drins might undergo biodilution during the growth of tilapia if the bioaccumulation rate of the pollutant is less than the growth rate^34^. A significant correlation was observed among HCH isomers, DDT isomers, and its metabolites. HCB was significantly related to DDTs, which suggested that they probably originated from similar contamination sources^23^. Similarly, CHLs and endosulfan probably came from similar sources.

In this study, higher correlations were found between contaminants and growth parameters than between contaminants and lipids, which may indicate that growth parameters are the predominant factor in determining OCs bioaccumulation^39,40^. This may be due to the fact that the intake of OCs in fish is closely related to their feeding intensity, and the feeding intensity increases with the length and weight of the fish^40^. Furthermore, exposure time (age of fish) is also a governing parameter in the uptake of contaminants. Considering the relatively higher correlation of OCPs and PCBs with total length and body mass rather than age (Table S7), we thought the food intake rate of fish better reflects the degree of pollution than age. After all, feeding is an important way for fish to be exposed to pollutants.

### Risk assessment of OCs via fish consumption

#### Estimated daily intakes (EDIs)

Based on the assessment method are shown in Text S4, the EDIs of OCPs through wild tilapia consumption from ten sites are summarized in Table S9. The assessment assumes a worst-case scenario for residents in the study area by using the highest measured concentrations in the calculation. The results of the study showed that the EDIs for males were almost equal to those for females, although previous studies reported significant differences^9^. For OCPs, dietary intakes from TD were 1.6 to 40 times higher than exposures from the other sites. The EDIs of all OCs through consumption of wild tilapia were far below the recommended acceptable daily intake (ADI)^41^, indicating minimum risk caused by these pollutants from tilapia consumption. However, fish and fish products account for higher than 10% of total food consumption in southern China^9^, so higher dietary intake of OCPs or PCBs can be expected when all food sources are taken into account.

#### Risk assessment

Relevant oral reference dose (RfD) values and cancer slope factors were used following the US Environmental Protection Agency Integrated Risk Information System (IRIS) (Table 2 and Text S5)^42^. Comparing the cancer benchmark concentrations derived from the main rivers in the southern Guangxi (this study) with those from the Chenab River, Pakistan^43^, almost all cancer benchmark concentrations for the residents in this study were lower than the Chenab River’s values. The primary reasons for this are the lower daily fish consumption rates and pollutant content in the southern Guangxi as compared to in Pakistan^2,9^.

**Table 2:** Maximum exposures and benchmark concentrations for contaminants in fish.

Compounds	Oral RfD^a^ (μg/kg/day)	Cancer slope factor^a^ (per μg/kg/day)	Cancer benchmark concentration (ng/kg/day)	50th MEC^b^ (ng/kg, ww)	95th MEC^b^ (ng/kg, ww)
Endosulfan	6			2.10E-03	7.91E-03
γ-HCH	0.3	1,100	0.87	1.71E-01	2.36E + 00
DDTs	0.5	340	2.81	1.81E-02	8.28E-02
Chlordane	0.06	350	2.73	9.60E-03	2.46E-02
Dieldrin	0.05	16,000	0.06	2.82E-02	6.73E-02
HCB	0.8	1,600	0.60	9.78E-02	1.33E-01
PCBs	0.02	2000	0.48	1.12E-01	7.58E + 00

^a^Oral RfDs and cancer slope factors were obtained from USEPA’s Integrated Risk Information System (IRIS).

^b^50th and 90th percentile MECs (measured concentrations) of OCPs and PCBs were used in the assessment of the risk posed to human health by the consumption of contaminated fish from the main river in the southern Guangxi, China.

**Figure 5:**
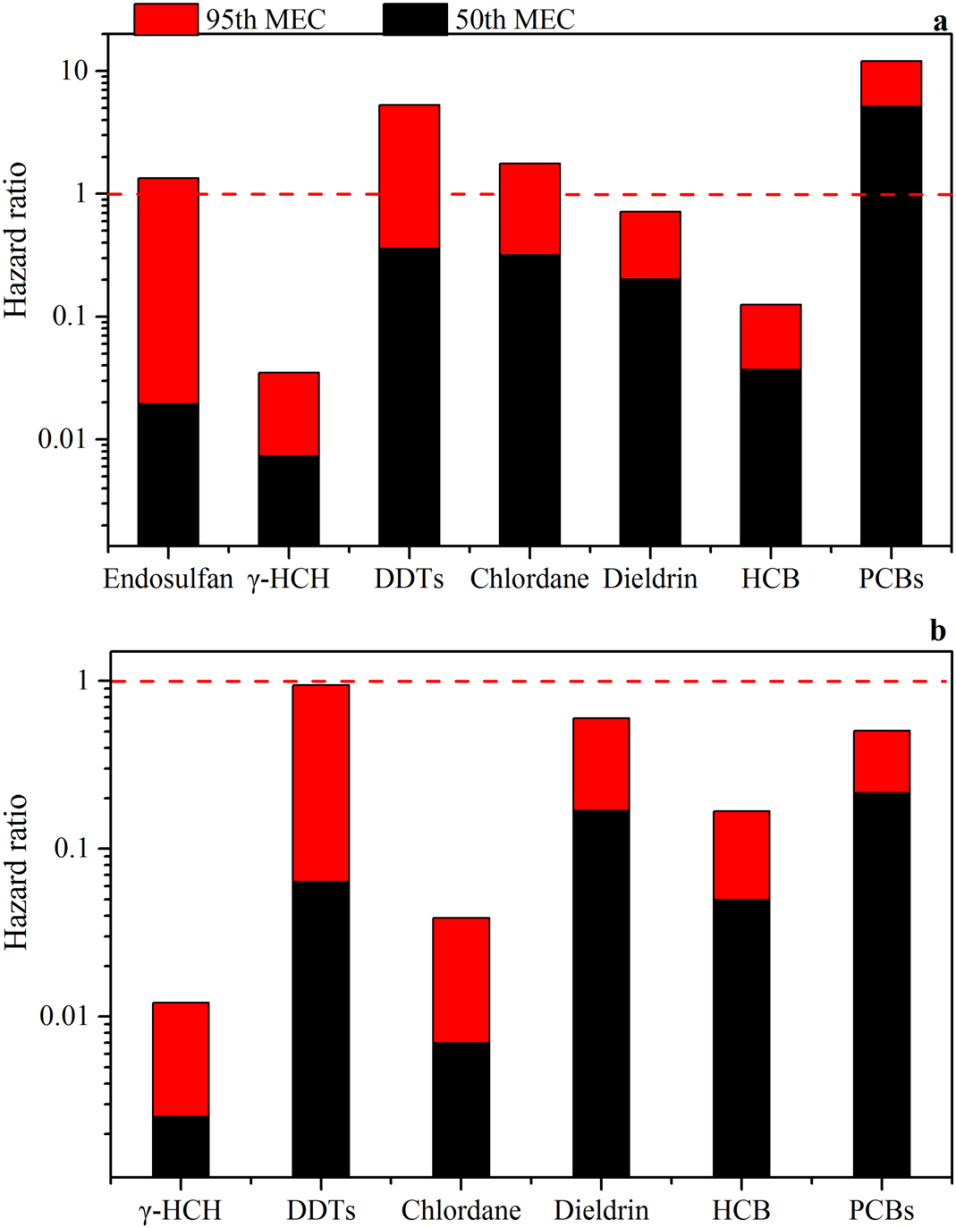
Hazard ratios for the daily consumption of wild tilapia from the main rivers in Guangxi, South China. (**a**) Non-cancer risks, (**b**) cancer risks. MEC: measured concentration.

Two HRs at the 50th and 95th percentiles were evaluated and summarized to assess both non-cancer and cancer risks associated with consumption of fish containing OC contaminants (Fig. 5). The HRs calculated for non-cancer risk assessment using the 50th and 95th percentiles of PCBs were greater than one, a result of the relatively high concentrations of PCBs in the fish samples collected from the main rivers in Guangxi, South China. The non-cancer risks based on 95th percentile concentrations of endosulfan, DDTs, and chlordane were also greater than one (Fig. 5 a). These results indicated that several compounds ingested via consumption of tilapia have a specific non-carcinogenic risk. On the other hand, the cancer risks associated with fish consumption based on 50th and 95th percentile concentrations of OC compounds were all less than one (Fig. 5 b), suggesting that daily exposure to these contaminants due to fish consumption has a lifetime cancer risk of less than one in one million. The cancer risk for DDTs (0.88) was found to be the highest among all organochlorines, followed by dieldrin (0.43), and PCB (0.29). Therefore, OCs do not present a lifetime cancer risk in this study, there is an increased risk for those who consume large quantities of wild tilapia.

There are several limitations in the present study. This investigation did not consider (1) potential risk based on different consumer groups and co-intake of many foods; (2) possible interactions among various toxic chemicals; (3) over-conservative risk assessment. Research on the influence of these limitations on the assessment results is in progress.

## Conclusions

To our knowledge, this study is the first time that the presence of PCBs and OCPs in wild tilapia from southern Guangxi have been reported. This study examined the level, distribution, and possible sources of 32 OC congeners. The organochlorine pollution of the freshwater ecosystem in the southern Guangxi was attributable to historical sources. However, recent applications of technical DDTs and endosulfan, and PCBs leakage in the study area should be given additional attention. Principal Component Analysis of biological parameters and OC compounds indicate that the feeding intensity of fish is the predominant factor in determining OCs bioaccumulation. A risk assessment concluded that exposure to OC compounds through tilapia consumption did not present a lifetime cancer risk. However, some factors, such as co-intake with other foods and large consumption of wild tilapia, are likely to result in some lifetime cancer risk to consumers.

## Supplementary information

Supplementary file1
