# Supplementary material for: Spatial distribution, source identification, and risk assessment of organochlorines in wild tilapia from Guangxi, South China
Source: Sci Rep. 2020 Sep 16;10:15179. doi: 10.1038/s41598-020-72160-x (PMC7495417; doi:10.1038/s41598-020-72160-x)
Supplement: Supplementary file 1 — Supplementary file1 [file 41598_2020_72160_MOESM1_ESM.pdf]

Spatial distribution, source identification, and risk assessment of organochlorines in wild tilapia from Guangxi, South China

Yang Ding[a,b], Yaru Kang[a,b], Zhiqiang Wu[c *], Ruijie Zhang[a,b**], Kefu Yu[a,b], Yinghui Wang[a,b], Xiaobo Zheng[d], Liangliang Huang [c], Lichao Zhao[a]

[a] School of Marine Sciences, Guangxi University, Nanning 530004, China

[b] Guangxi Laboratory on the Study of Coral Reefs in the South China Sea, Coral Reef Research Center of China, Guangxi University, Nanning 530004, China

[c] College of Environmental Science and Engineering, Guilin University of Technology, Guilin, Guangxi, China

[d] College of Resources and Environment, South China Agricultural University, Guangzhou 510642, China

*** Corresponding author:** Zhiqiang Wu, Guilin University of Technology, Guilin 541004, China. E-mail: wuzhiqiang@glut.edu.cn.

**** Correspondence author:** Ruijie Zhang, School of Marine Sciences, Guangxi University, Nanning 530004, China. E-mail: rjzhang@gxu.edu.cn.

Contents

Text S1. Study area.....3

Text S2 Chemicals.....4

Text S3 QA/QC.....4

Text S4 Calculation of Estimated Daily Intakes (EDIs).....5

Text S5 Risk assessment.....6

Table S1. Biological parameters of fish samples.....8

Table S2. Physicochemical characteristics, standards producers of the target OCs and surrogates.....10

Table S3 Limit of detection (LOD) and limit of quantification (LOQ) for the OCs chemicals.....12

Table S4. Concentration levels of 7 OC compounds in 75 tilapia samples (ng/g lw).....13

Table S5. Comparison of concentrations of OCs in tilapia worldwide.....15

Table S6. Ratios of OCs for source identification in wild tilapia......16

Table S7. Correlation coefficient matrix for OC compounds (based on wet weight) and growth parameters of fish samples (n = 75).....18

Table S8. Correlation coefficient matrix for OC compounds and growth parameters of fish samples (n = 75). Correlation coefficient matrix for individual OCs and growth parameters of fish samples (n = 75)......19

Table S9. Estimated daily intakes (EDIs) of OCPs and PCBs in fish by humans using maximum concentrations of OCPs and PCBs (ng/g, ww) in collected wild tilapia. ...21

Fig. S1. Studied wild tilapias were purchased from the main river in Guangxi, South China22

Fig. S2**.** Distribution map of sampling locations in the main river in Guangxi, South China. TD: Tiandong County; LA: Longan County; CZ: Chongzuo City; FS: Fusui City; NN: Nanning City; GG: Guigang City; WX: Wuxuan County; PN: Pingnan City; TX: Tengxian County; WZ: Wuzhou City**.**.....23

Text S1. Study area

Five ecologically important main rivers were selected from the southern of Guangxi province, including Youjiang River (YO), Zuojiang River (ZU), Yujiang River (YU), Qianjiang River (QI) and Xunjiang River (XU) (Fig. 1). With a total drainage area of approximately 175,412 square kilometers, it is the most important water system in Guangxi. The southern river system of Guangxi has become an important resource support and pollution bearer for regional economic development. The basin is home to more than 73.4% of the province's industrial enterprises, and is a veritable industrial area, yet until recently their ecological condition has not been well quantified. This basin is also China's largest sugarcane producing area (about 52.1% of the country's total planting area), and is also a high incidence area for pests and diseases (Guangxi Statistics Bureau, 2019). In order to control pests and diseases, a large number of chemical pesticides have been used for a long time. In 2015-2018, the average annual use of pesticides in Guangxi was 13,800 tons (Guangxi Statistics Bureau, 2019). The escalating anthropogenic activities (agricultural run-off, industrial waste and municipal discharge) along the basin may seriously pollute the region with OCs and may cause high accumulations of OCs in the riverine biota. Many studies have reported the concentration of POPs in their downstream water bodies (Xijiang River Basin and Pearl River Delta) (Mai, et al., 2005; Guo, et al., 2008; Qiao, et al., 2010; Sun, et al., 2015), but studies on the concentration of POPs in the southwestern Guangxi have not been reported.

Text S2 Chemicals

Fifty-three OCs congeners were selected as target compounds. The OCPs were dicided into seven groups including DDTs, HCHs, Drins, CHLs, Endos, HCB and MXC, The 28 PCBs from the groups of tri-, tetra-, penta-, hexa-, hepta-CBs were selected as target compounds in this study. Physicochemical properties, purchasing channels and handling procedures of all the target compounds and chemicals are listed in Table S2.

All the target OCs and surrogates standards were high purity more than 99%. The three recovery indicators (2.4.5.6-tetrachloro-m-xylene (TCMX), PCB30, PCB204) and internal standard (^{13}C12-PCB138) of OCPs and PCBs were purchased from Accustandard (America). All solvents used for the analysis were HPLC-grade. Dichloromethane (DCM) and n-hexnane (HEX) were purchased from CNW (ANPEL Laboratory Technologies (Shanghai) co. Ltd). Aceton were purchased from KNOWLES (Chengdu chren chemical co. Ltd). Deactivated neutral alumina: 70-230 mesh (Merck), extracted using DCM for 48 h, roasted in an oven at 250 ℃ for 12 h and burned in a muffle furnace at 450 ℃ for 6 h, added with 3% distilled water after cooling to activate; Acid silical gel: 80-200 mesh (Merck), extracted using DCM for 48 h, roasted in an oven at 180 ℃ for 12 h and burned in a muffle furnace at 450 ℃ for 6 h; added with sulfuric acid accounting for half of the total weight after cooling; Anhydrous anhydrous sodium sulfate was burned in a muffle furnace at 450 ℃ for 6 h. All of the three reagents was placed in a desiccator before use. Ultra-pure water was prepared with a Milli-Q water-purification system (Millipore, Bedford, Massachusetts, USA). ENVI™-Florisil cartridges (500 mg, 3 mL) were purchased from Supleco (Bellefonte, PA, USA).

Text S3 QA/QC

The standard solutions with nine concentration gradients (concentration range was between 1–500 ng/mL) were detected and analyzed, and the regression coefficient of > 0.97. Each sample was analyzed three times and the average was used for data

analysis. A standard solution (2 ng/mL for each OC compounds) with a fixed concentration was injected every day to monitor the sensitivity of the instrument. Procedural blanks (n = 8), spiked blanks (n = 8), and spiked matrices (n = 8) were set to ensure method quality control. The laboratory procedural blanks were analyzed using extracted organism samples (fish muscle). No targeted OC congeners were detected in the procedural blank. The spiked compounds included 24 OCP congeners and 28 PCB congeners. The mean recoveries of the surrogates in all samples were 72.3 ± 8.4%, 87.6 ± 20.0%, and 92.4 ± 17.8% for TCMX, PCB30, and PCB204, respectively. The concentrations reported in this paper were after subtracting the mean concentration in the blanks analyzed in the same batch, but not adjusted by surrogate recoveries. Recovery of OC standards was 68.6–96.2% in the spiked blanks and 73.8–105.6% in the matrix-spiked samples, with a relative standard deviation of < 17%. Instrumental detection limits (IDLs) and instrumental quantification limits (IQLs) were defined as three and ten times the signal-to-noise (S/N) ratio, respectively. The LODs and LOQs for the tilapia samples were 0.006–0.087 ng/g lw (lipid weight) and 0.020–0.289 ng/g lw (Table S3).

Text S4 Calculation of Estimated Daily Intakes (EDIs).

EDI values for the OC compounds (Table GG) were calculated using the following equation:

$$\mathrm{EDI} = \frac{\mathrm{PR} \times \mathrm{C}}{\mathrm{BW}}$$

Where EDI is the estimated daily intake of the OC compounds in ng/kg body weight (bw)/day (d); PR is the annual per capita fish consumption of 59.3 g/person/day in Southern China (Guo, et al., 2010); C is the average concentration of PCBs and OCPs in fish muscle (ng/g wet weight (ww)); BW is the average body weight of 56.6 kg (Ding, et al., 2019); C is selected the maximum concentration of each OC compounds (ng/g

ww) detected in wild tilapia from the main river in the southern China.

Text S5 Risk assessment.

Hazard ratios (HRs) were computed to assess risk associated with fish consumption in humans (Dougherty, et al., 2000; Jiang, et al., 2005). Hazard ratios (HRs) were calculated by dividing the estimated daily intakes (EDIs) by the benchmark concentrations (BMC). There would be no obvious hazard if HRs is less than 1 (Jiang, et al., 2005). The hazard ratios were assessed by the equation:

$$\text{HRs} = \frac{EDIs}{BMC}$$

The benchmark concentration (BMC) for carcinogenic effects consists of two components: (1) the cancer slope factor (CSF, mg/kg/d); (2) the non-carcinogenic effects, based on the oral reference dose (RfD, mg/kg/d), obtained from the US Environmental Protection Agency (USEPA) Integrated Risk Information System (IRIS) for each contaminant. For carcinogenic effects, cancer benchmark concentration (CMC) calculated by using equation below:

$$\text{CMC} = \frac{RL \times BW}{CSF \times CR}$$

Where RL is the maximum acceptable risk level (1×10^{-6}); CSF is the cancer slope factor (mg/kg/d); CR is the consumption rate (g/d).

Two hazard ratios (HRs), i.e., from the 50th and the 95th percentile measured concentrations (50th and 95th MEC), were used for assessing the potential health risk to humans. The two HRs provided a simple way for screening chemicals that might require a more detailed analysis. When 95th centile HR was greater than unity, a refined

risk assessment would subsequently be conducted to further ascertain the real risk. When both 50th and 95th centile HRs were greater than unity, an initiation of appropriate management strategies may be considered (USEPA, 2000; Jiang, et al., 2005).

Table S1. Biological parameters of fish samples

Sample site	Sample code	Total length (cm)	Body length (cm)	Body mass (g)	Age (year)	Water contents (%)	Lipid (%)[a]
TD	TD-NL[b]-1	178.1	139.1	138.9	0	79.7%	5.37%
	TD-NL-2	180.7	142.8	134.4	0	79.7%	5.36%
	TD-NL-3	206.4	161.9	178.9	1	79.7%	4.58%
	TD-NL-4	189.8	148.1	145.8	1	79.7%	6.08%
	TD-NL-5	216.9	169.4	230.6	1	79.7%	5.80%
	TD-RB[c]-1	159.7	126.4	91.9	0	80.4%	5.70%
	TD-RB-2	143.6	113.0	65.4	1	80.4%	6.08%
	TD-RB-3	175.1	139.9	130.2	1	80.4%	5.92%
	TD-RB-4	168.0	132.4	104.6	0	80.4%	6.78%
	TD-RB-5	181.7	144.7	142.0	1	80.4%	5.72%
LA	LA-NL-1	181.4	143.4	129.6	2	78.8%	3.96%
	LA-NL-2	175.5	135.7	109.7	1	78.8%	3.41%
	LA-NL-3	184.7	145.2	131.2	1	78.8%	3.69%
CZ	CZ-NL-1	169.7	133.3	96.4	1	81.1%	6.86%
	CZ-RB-1	174.5	137.6	112.4	1	79.6%	5.83%
	CZ-RB-2	194.3	152.8	150.3	1	79.6%	4.27%
	CZ-RB-3	179.8	142.5	127.7	1	79.6%	5.05%
	CZ-RB-4	188.0	150.3	144.2	1	79.6%	4.73%
	CZ-RB-5	174.7	136.9	124.1	1	79.6%	4.85%
	CZ-RB-6	204.5	160.0	149.5	2	79.6%	4.61%
	CZ-RB-7	161.2	128.9	84.7	1	79.6%	5.16%
FS	FS-NL-1	103.1	78.4	20.7	1	80.5%	5.20%
	FS-NL-2	183.1	144.6	125.5	1	80.5%	4.97%
	FS-NL-3	184.5	144.5	155.6	1	80.5%	5.07%
	FS-NL-4	172.1	135.1	119.8	1	80.5%	4.69%
	FS-RB-1	178.8	141.4	113.8	1	78.3%	2.08%
	FS-RB-2	181.3	147.5	135.1	2	78.3%	4.61%
	FS-RB-3	161.9	127.5	82.9	1	78.3%	3.89%
NN	NN-NL-1	19.6	16.1	137.1	1	80.6%	2.75%
	NN-NL-2	18.4	14.7	123.7	1	79.7%	4.00%
	NN-NL-3	16.5	12.7	97.5	1	80.5%	3.59%
	NN-RB-1	19.0	15.4	136.3	1	80.9%	2.57%
	NN-RB-2	19.0	15.2	147.5	1	79.6%	7.23%
	NN-RB-3	21.3	17.1	206.1	2	77.7%	4.24%
	NN-RB-4	18.1	14.9	147.0	1	76.9%	5.71%
	NN-RB-5	16.4	13.4	89.3	1	79.1%	5.83%
	NN-RB-6	20.4	16.6	175.8	2	79.4%	4.05%
GG	GG-NL-1	228.3	167.6	265.8	2	80.1%	7.49%

	GG-NL-2	224.3	178.2	218.2	1	79.3%	7.79%
	GG-NL-3	246.3	188.9	344.7	2	80.3%	5.87%
	GG-NL-4	213.6	172.5	205.7	2	78.7%	7.13%
	GG-NL-5	228.5	175.6	214.9	2	78.8%	9.95%
	GG-RB-1	147.7	115.0	72.0	1	77.7%	10.8%
	GG-RB-2	169.3	130.5	115.6	1	78.3%	6.77%
	GG-RB-3	147.5	116.6	76.5	1	78.7%	5.72%
	GG-RB-4	151.6	119.0	83.2	2	76.0%	18.7%
WX	WX-NL-1	134.6	108.7	53.2	0	80.4%	3.86%
	WX-NL-2	133.2	101.6	51.8	0	80.4%	3.73%
	WX-NL-3	153.3	119.6	92.4	1	80.4%	3.41%
	WX-NL-4	122.5	95.4	39.2	0	80.4%	3.58%
	WX-NL-5	145.9	117.4	74.9	1	80.4%	4.65%
PN	PN-NL-1	186.3	145.5	131.2	1	77.7%	9.43%
	PN-NL-2	235.3	180.2	257.3	2	75.2%	17.0%
	PN-NL-3	180.0	137.5	121.5	1	77.7%	7.95%
	PN-NL-4	231.2	179.2	261.3	2	76.3%	14.2%
	PN-NL-5	195.3	149.5	174.0	1	78.5%	12.1%
	PN-RB-1	128.8	102.9	42.3	1	79.3%	4.60%
	PN-RB-2	132.7	106.4	47.3	1	79.0%	6.67%
	PN-RB-3	171.5	135.5	91.1	1	79.0%	6.29%
	PN-RB-4	142.0	110.8	57.0	1	78.3%	6.19%
	PN-RB-5	136.5	109.3	47.4	1	79.7%	7.51%
TX	TX-NL-1	165.0	130.4	80.7	1	78.6%	3.90%
	TX-NL-2	132.8	105.1	45.9	1	78.6%	5.71%
	TX-NL-3	234.5	192.2	317.1	1	78.7%	4.26%
	TX-NL-4	241.3	192.6	296.5	1	79.2%	4.19%
	TX-NL-5	233.5	182.6	263.9	1	78.3%	2.50%
	TX-RB-1	126.1	96.5	38.0	1	81.4%	2.22%
	TX-RB-2	131.9	106.4	54.9	1	81.5%	2.28%
	TX-RB-3	134.8	105.2	49.8	1	81.5%	2.88%
	TX-RB-4	155.3	121.0	69.4	1	81.5%	2.35%
WZ	WZ-NL-1	308.1	240.2	614.0	3	80.4%	4.63%
	WZ-NL-2	293.3	229.4	582.9	2	80.9%	3.80%
	WZ-NL-3	290.6	233.5	584.3	2	80.3%	5.33%
	WZ-NL-4	286.8	226.3	522.6	1	79.8%	6.84%
	WZ-NL-5	161.4	122.5	90.4	1	79.8%	4.86%

[a] Based on dry weight

[b] NL: nile tilapia (*Oreochromis niloticus*, (Linnaeus, 1758));

[c] RB: redbelly tilapia (*Coptodon zillii*, (Gervais, 1848))

Table S2. Physicochemical characteristics, standards producers of the target OCs and surrogates

OCs (Abbreviation)	CAS NO[a]	Molecular formula[a]	Molecular weight [b]	Water Solubility [b]	Log Kow [b]	Standards supplier [c]	vapor pressure[b]	Henry's law constant[b]
α-HCH	319-84-6	$C_6H_6Cl_6$	291	2.68E-05	3.72	1	5.57E-03	7.34E-06
β-HCH	319-85-7	$C_6H_6Cl_6$	292	2.68E-05	3.72	1	6.97E-03	7.34E-06
γ-HCH	58-89-9	$C_6H_6Cl_6$	293	2.68E-05	4.14	1	6.97E-03	7.34E-06
δ-HCH	319-86-8	$C_6H_6Cl_6$	294	2.17E-05	3.72	1	6.97E-03	7.34E-06
o,p'-DDT	789-02-6	$C_{14}H_9Cl_5$	354	3.73E-07	6.79	1	2.15E-06	1.46E-05
p,p'-DDT	50-29-3	$C_{14}H_9Cl_5$	354	2.38E-07	6.91	1	1.10E-06	1.05E-05
o,p'-DDE	3424-82-6	$C_{14}H_8Cl_4$	318	5.58E-07	6	1	9.22E-06	1.17E-05
p,p'-DDE	72-55-9	$C_{14}H_8Cl_4$	318	2.68E-07	6.51	1	1.08E-05	2.68E-05
o,p'-DDD	53-19-0	$C_{14}H_{10}Cl_4$	320	5.24E-07	5.87	1	3.72E-06	1.60E-05
p,p'-DDD	72-54-8	$C_{14}H_{10}Cl_4$	320	4.14E-07	6.02	1	3.39E-06	1.01E-05
TC	5103-74-2	$C_{10}H_6Cl_8$	410	1.19E-07	6.16	1	1.15E-05	2.00E-04
CC	5103-71-9	$C_{10}H_6Cl_8$	411	1.16E-05	6.16	1	1.19E-07	2.00E-04
Heptachlor	76-44-8	$C_{10}H_5Cl_7$	373	7.86E-06	5.4	1	7.65E-07	2.58E-05
Heptachlor epoxide(B)	1024-57-3	$C_{10}H_5Cl_7O$	389	3.99E-07	5.4	1	1.45E-05	2.58E-05
Heptachlor epoxide(A)	28044-83-9	$C_{10}H_5Cl_7O$	389	7.86E-06	5.47	1	7.65E-07	2.58E-05
α-Endosulfan	959-98-8	$C_9H_6C_{l6}O_3S$	407	4.23E-06	3.83	1	3.77E-07	2.54E-05
β-Endosulfan	33213-65-9	$C_9H_6C_{l6}O_3S$	407	4.25E-06	3.83	1	4.06E-07	2.54E-05
Endosulfan sulfate	1031-07-8	$C_9H_6C_{l6}O_4S$	422.9	2.80E-06	3.64	1	2.11E-07	1.14E-05
Aldrin	309-00-2	$C_{12}H_8Cl_6$	365	2.25E-04	6.5	1	8.91E-08	5.18E-05
Dieldrin	60-57-1	$C_{12}H_8Cl_6O$	381	2.43E-06	5.6	1	1.40E-06	1.30E-05
Endrin	72-20-8	$C_{12}H_8Cl_6O$	381	2.43E-06	5.4	1	1.38E-06	1.30E-05
Endrin aldehyde	7421-93-4	C12H8Cl6O	381	9.03E-08	5.63	1	4.35E-07	1.09E-05
Endrin ketone	53494-70-5	$C_{12}H_8Cl_6O$	347	2.44E-07	5.61	1	3.12E-06	5.65E-05
HCB	118-74-1	C_6Cl_6	285	4.08E-07	5.73	1	5.01E-04	1.20E-03
MXC	72-43-5	$C_{16}H_{15}Cl_3O_2$	346	1.25E-06	5.08	1	1.28E-06	2.86E-07
PCB8	34883-43-7	$C_{12}H_8Cl_2$	223.1	4.79E-06	5.05	1	1.49E-03	2.50E-04
PCB18	37680-65-2	$C_{12}H_7Cl_{13}$	258	1.64E-06	5.5	1	8.95E-04	2.18E-04
PCB28	7012-37-5	$C_{12}H_7Cl_{13}$	258	1.15E-06	5.61	1	1.29E-03	1.94E-04
PCB52	35693-99-3	$C_{12}H_6Cl_{14}$	292	3.63E-07	5.94	1	6.97E-05	1.78E-04

PCB44	41464-39-5	$C_{12}H_6Cl_{14}$	292	3.15E-07	5.88	1	6.32E-05	1.29E-04
PCB66	32598-10-0	$C_{12}H_6Cl_{14}$	292	2.47E-07	6.05	1	6.06E-05	1.04E-04
PCB81	70362-50-4	$C_{12}H_6Cl_{14}$	292	3.74E-07	6.01	1	7.02E-05	5.55E-05
PCB77	32598-13-3	$C_{12}H_6Cl_{14}$	292	4.25E-07	6	1	1.30E-04	3.57E-05
PCB101	37680-73-2	$C_{12}H_5Cl_{15}$	326	7.62E-08	6.38	1	1.99E-05	8.39E-05
PCB123	65510-44-3	$C_{12}H_5Cl_{15}$	326	1.45E-07	6.39	1	1.98E-05	7.23E-05
PCB126	31508-00-6	$C_{12}H_5Cl_{15}$	326	7.20E-08	6.42	1	1.91E-05	7.87E-05
PCB118	74472-37-0	$C_{12}H_5Cl_{15}$	326	9.36E-08	6.44	1	1.70E-05	5.72E-05
PCB114	32598-14-4	$C_{12}H_5Cl_{15}$	326	1.14E-07	6.39	1	1.75E-05	5.65E-05
PCB105	57465-28-8	$C_{12}H_5Cl_{15}$	326	1.66E-07	6.83	1	2.47E-05	5.92E-05
PCB153	35065-27-1	$C_{12}H_4Cl_{16}$	361	1.15E-08	6.83	1	4.97E-06	2.93E-05
PCB138	35065-28-2	$C_{12}H_4Cl_{16}$	361	2.52E-08	6.87	1	5.90E-06	2.40E-05
PCB169	38380-07-3	$C_{12}H_4Cl_{16}$	361	1.82E-08	7.12	1	5.49E-06	2.04E-05
PCB128	52663-72-6	$C_{12}H_4Cl_{16}$	361	1.95E-08	6.89	1	4.29E-06	2.20E-05
PCB167	38380-08-4	$C_{12}H_4Cl_{16}$	361	3.47E-08	6.89	1	5.46E-06	2.85E-05
PCB156	69782-90-7	$C_{12}H_4Cl_{16}$	361	4.64E-08	7.14	1	5.45E-06	2.85E-05
PCB157	32774-16-6	$C_{12}H_4Cl_{16}$	361	5.74E-08	7.16	1	9.40E-06	2.94E-05
PCB180	52663-68-0	$C_{12}H_3Cl_{17}$	395	5.71E-09	7.12	1	2.07E-06	1.31E-05
PCB187	35065-29-3	$C_{12}H_3Cl_{17}$	395	7.65E-09	7.16	1	1.48E-06	7.30E-06
PCB170	35065-30-6	$C_{12}H_3Cl_{17}$	395	1.04E-08	7.11	1	1.87E-06	1.13E-05
PCB189	39635-31-9	$C_{12}H_3Cl_{17}$	395	1.27E-08	7.2	1	2.88E-06	8.32E-06
PCB195	52663-78-2	$C_{12}H_2Cl_8$	429.75	3.46E-10	7.44	1	8.91E-07	1.09E-05
PCB206	40186-72-9	$C_{12}HCl_9$	464.19	6.76E-11	7.81	1	5.41E-08	1.09E-05
PCB209	2051-24-3	$C_{12}Cl_{10}$	498.662	7.18E-11	8.3	1	5.78E-08	1.09E-05

[a] Data are from U.S. National Library of Medicine ChemnIDPlus Advanced (https://chem.nlm.nih.gov/chemidplus/). [b] Data are from (https://comptox.epa.gov/dashboard/dsstoxdb/). [c] Standards supplier: 1 Wellington Laboratories (USA)

Table S3 Limit of detection (LOD) and limit of quantification (LOQ) for the OCs chemicals

Chemicals	SNR	LOD	LOQ	Chemicals	SNR	LOD	LOQ
α-HCH	140.9	0.023	0.075	PCB28	336.3	0.009	0.031
β-HCH	69.5	0.045	0.149	PCB44	327.6	0.010	0.032
γ-HCH	128.0	0.024	0.081	PCB52	222.6	0.014	0.048
δ-HCH	122.9	0.026	0.087	PCB66	264.4	0.012	0.038
DDE-o,p'	392.4	0.008	0.026	PCB77	118.8	0.026	0.087
DDE-p,p'	471.5	0.006	0.022	PCB81	118.8	0.027	0.088
DDD-o,p'	187.6	0.017	0.056	PCB101	190.8	0.017	0.055
DDD-p,p'	106.8	0.029	0.096	PCB105	77.2	0.041	0.136
DDT-o,p'	106.8	0.029	0.098	PCB114	115.2	0.027	0.089
DDT-p,p'	126.9	0.025	0.083	PCB118	115.2	0.027	0.091
MXC	96.4	0.032	0.106	PCB123	115.2	0.027	0.091
TC	105.8	0.030	0.100	PCB126	162.8	0.019	0.063
CC	137.1	0.022	0.075	PCB128	132.3	0.022	0.075
Heptachlor	53.9	0.059	0.198	PCB138	523.4	0.006	0.020
Heptachlor epoxide (A)	168.2	0.020	0.065	PCB153	216.4	0.014	0.047
Heptachlor epoxide (B)	196.5	0.017	0.058	PCB156	269.3	0.011	0.038
α-Endosulfan	36.6	0.074	0.246	PCB157	194.6	0.016	0.053
β-Endosulfan	95.3	0.033	0.109	PCB167	158.7	0.019	0.064
Endosulfan sulfate	330.2	0.009	0.031	PCB169	225.8	0.013	0.043
Aldrin	66.3	0.049	0.164	PCB170	176.4	0.017	0.056
Endrin	38.0	0.073	0.245	PCB180	443.5	0.007	0.022
Dieldrin	35.5	0.087	0.289	PCB187	283.2	0.010	0.034
Endrin aldehyde	106.8	0.030	0.101	PCB189	154.1	0.018	0.061
Endrin ketone	50.4	0.063	0.211	PCB195	349.9	0.008	0.027
HCB	504.2	0.006	0.021	PCB206	140.9	0.022	0.072
PCB8	166.8	0.019	0.063	PCB209	193.0	0.016	0.053
PCB18	370.3	0.009	0.029				

Table S4. Concentration levels of 7 OC compounds in 75 tilapia samples (ng/g lw)

Site	Code	HCHs [a]	DDTs [b]	CHLs [c]	Endosulfand	Drins [e]	HCB	OCP	PCBs [f]
S1	S1-NL-1	0.47	8.60	3.47	685	7.09	1.52	706	9.34
	S1-NL-2	0.72	7.87	3.42	674	4.73	1.23	692	7.04
	S1-NL-3	0.53	7.89	7.73	717	8.21	1.14	742	12.2
	S1-NL-4	0.59	4.50	3.10	385	5.32	1.45	399	8.13
	S1-NL-5	0.98	7.23	1.76	609	4.56	0.86	624	8.38
	S1-RB-1	0.81	10.2	4.61	754	3.90	1.47	775	8.93
	S1-RB-2	0.93	9.9	7.04	925	4.46	1.77	949	9.63
	S1-RB-3	0.72	6.53	9.11	629	6.34	1.84	653	8.37
	S1-RB-4	0.84	7.31	8.79	625	4.89	1.18	648	7.48
	S1-RB-5	0.71	6.54	8.41	627	3.71	1.21	648	7.54
S2	S2-NL-1	1.08	17.5	3.11	13.3	10.2	2.47	47.7	10.5
	S2-NL-2	1.41	18.8	2.52	22.3	7.89	2.05	55.0	11.4
	S2-NL-3	1.05	22.5	10.8	8.58	13.36	2.36	58.7	11.9
S3	S3-NL-1	1.28	38.3	1.19	15.6	6.65	3.86	66.9	7.35
	S3-RB-1	1.37	30.6	1.16	11.9	7.02	3.21	55.2	8.41
	S3-RB-2	1.43	13.8	1.81	12.9	9.83	2.72	42.5	10.6
	S3-RB-3	0.97	13.0	2.81	9.25	10.7	2.16	38.9	9.35
	S3-RB-4	1.25	11.0	1.41	6.61	7.74	2.90	30.9	11.4
	S3-RB-5	1.27	11.0	1.36	9.80	7.29	3.11	33.9	11.3
	S3-RB-6	0.79	13.3	2.64	11.9	5.69	3.24	37.6	9.31
	S3-RB-7	1.24	8.95	1.24	8.54	5.98	2.94	28.9	9.41
S4	S4-NL-1	1.35	16.0	2.17	9.84	10.1	2.95	42.4	10.7
	S4-NL-2	1.42	11.0	2.43	6.38	9.85	3.12	34.2	8.59
	S4-NL-3	1.14	12.4	2.11	8.59	8.30	2.36	34.9	9.68
	S4-NL-4	1.51	14.5	1.91	10.2	6.13	3.80	38.0	9.81
	S4-RB-1	2.26	5.98	3.83	9.46	12.8	8.21	42.6	29.3
	S4-RB-2	0.82	9.71	3.47	13.4	5.64	2.12	35.1	8.34
	S4-RB-3	1.28	10.1	3.27	36.4	8.58	2.89	62.5	11.2
S5	S5-NL-1	1.77	11.5	1.74	10.9	11.3	3.76	41.0	15.67
	S5-NL-2	0.83	6.41	2.11	8.11	6.40	3.78	27.6	9.11
	S5-NL-3	1.14	4.57	1.62	8.13	12.3	3.15	30.9	9.83
	S5-RB-1	1.71	19.3	6.34	8.92	20.0	5.35	61.5	17.6
	S5-RB-2	0.48	15.5	1.52	9.38	2.83	3.29	33.0	6.64
	S5-RB-3	0.70	6.54	0.90	6.88	10.1	2.00	27.1	6.56
	S5-RB-4	1.21	8.35	1.36	15.2	6.45	3.18	35.7	7.81
	S5-RB-5	0.62	15.7	0.98	11.0	3.75	2.79	34.9	8.59
	S5-RB-6	0.88	26.4	1.48	9.82	6.13	1.71	46.4	12.7
S6	S6-NL-1	3.15	253	1.23	14.2	5.24	4.12	281	10.5
	S6-NL-2	3.40	422	2.15	16.2	4.73	3.79	452	8.58
	S6-NL-3	3.42	155	1.63	13.2	5.31	4.19	183	11.9

	S6-NL-4	2.72	238	0.59	7.76	3.83	3.36	257	8.13
	S6-NL-5	3.50	339	0.87	11.7	7.33	3.28	365	8.79
	S6-RB-1	2.26	32.0	1.01	17.5	2.86	2.95	58.6	5.17
	S6-RB-2	1.73	39.1	1.10	14.9	7.77	2.67	67.2	6.09
	S6-RB-3	2.93	33.8	1.93	17.6	10.5	4.65	71.5	8.41
	S6-RB-4	2.62	20.1	0.95	17.6	1.95	2.80	46.0	3.36
S7	S7-NL-1	1.40	8.43	2.40	8.84	12.3	1.38	34.7	6.90
	S7-NL-2	1.23	12.0	2.80	6.08	10.8	1.60	34.4	9.46
	S7-NL-3	1.17	14.0	3.14	10.7	10.8	3.95	43.7	12.5
	S7-NL-4	0.83	15.4	2.39	5.20	11.9	2.08	37.8	15.2
	S7-NL-5	0.81	9.98	2.46	6.48	4.50	1.41	25.6	8.11
S8	S8-NL-1	1.66	11.7	1.10	9.63	4.66	2.49	31.2	6.53
	S8-NL-2	1.45	6.66	0.43	10.7	2.11	2.58	23.9	3.44
	S8-NL-3	1.41	5.06	1.49	8.04	5.35	3.17	24.5	4.50
	S8-NL-4	1.71	8.34	0.65	9.34	2.51	2.24	24.8	3.49
	S8-NL-5	1.51	6.85	0.77	7.31	4.20	2.69	23.3	3.98
	S8-RB-1	2.19	58.3	1.78	12.7	13.0	5.65	93.6	13.5
	S8-RB-2	2.34	35.6	1.87	13.4	3.63	4.96	61.8	6.80
	S8-RB-3	1.65	68.9	0.79	7.38	5.79	4.88	89.4	8.49
	S8-RB-4	1.35	54.9	0.99	6.83	5.48	4.33	73.9	7.03
	S8-RB-5	1.30	24.8	0.54	7.90	3.55	2.71	40.8	8.57
S9	S9-NL-1	1.39	50.3	2.25	4.56	4.90	3.53	66.9	11.4
	S9-NL-2	0.99	35.2	1.40	2.25	4.78	1.63	46.3	7.59
	S9-NL-3	0.94	31.4	1.81	7.21	5.59	2.36	49.3	9.83
	S9-NL-4	2.99	44.3	1.81	3.33	4.25	3.13	59.8	14.4
	S9-NL-5	1.88	67.1	2.75	9.22	15.1	4.00	100	17.1
	S9-RB-1	2.12	56.3	1.33	3.66	11.0	3.16	77.6	18.6
	S9-RB-2	1.95	88.9	2.04	3.02	12.2	6.00	114	19.9
	S9-RB-3	2.46	49.0	1.54	10.6	6.35	3.81	73.8	17.8
	S9-RB-4	3.49	71.5	1.05	3.25	13.8	2.83	95.9	19.3
S10	S10-NL-1	5.19	22.0	1.60	10.6	8.14	5.47	53.0	10.9
	S10-NL-2	3.63	25.9	3.17	7.34	5.47	4.95	50.4	9.84
	S10-NL-3	4.99	17.6	2.04	22.0	3.42	3.53	53.6	7.23
	S10-NL-4	5.61	15.2	2.02	20.9	3.18	5.56	52.5	6.33
	S10-NL-5	2.11	6.65	1.24	9.18	11.0	0.71	30.9	2.65

[a] Sum of α-HCH, β-HCH, γ-HCH and δ-HCH; [b] Sum of o,p', p,p'-DDD, -DDE, -DDT; [c] Sum of TC, CC, heptachlor endo-epoxide; [d] Sum ofα-, β-endosulfan, and endosulfan sulfate; [e] Sum of Aldrin, Endrin, Dieldrin, Endrin aldehyde and Endrin ketone; [f] Sum of CB-8, 18, 28, 44, 52, 101, 189, 195, 206, 209.

Table S5. Comparison of concentrations of OCs in tilapia worldwide

Study area	HCHs	DDTs	CHLs	HCB	Endosulfan	PCBs	Unit	Reference
Fish ponds (China)	/[a]	10.62	/	/	/	/	ng/g ww	(Kong et al., 2005)
Guangdong (China)	/	32.2	/	/	/	/	ng/g lw	(Guo et al., 2010)
Pearl River Delta (China)	5.5	380	/	/	/	37	ng/g lw	(Sun, 2016)
Burullus Lake (Egypt)	1.88	9.85	/	/	/	18.62	ng/g ww	(Said et al., 2008)
Victoria Lake (Uganda)	2.4	13.6	1.4	0.14	2.1	/	ng/g lw	(Ogwok et al., 2009)
Tanganyika Lake (Burundi)	0.57	393	/	6.5	0.5	106.4	ng/g lw	(Manirakiza et al., 2002)
Babati Lake (Tanzania)	/	42.7	0.1	1.2	1.2	0.1	ng/g lw	(Polder et al., 2014)
Weija Lake (Ghana)	0.72	440.9	7.19	2.1	/	1.2	ng/g lw	(Adu-Kumi et al., 2010)
Danube delta (Romania)	180	215	/	4.7	/	134	ng/g lw	(Covaci et al., 2006)
the Salton Sea (USA)	0.3	15	/	2.6	/	20.54	ng/g ww	(Sapozhnikova et al., 2004)
Brazil	0.38	0.53	/	<0.01	1.58	/	ng/g ww	(Botaro et al., 2011)
The main rivers (South China)	1.37 (0.02)	15.2 (0.51)	1.81 (0.02)	2.94 (0.03)	10.2 (1.15)	9.11 (0.10)	ng/g lw (ng/g ww)	This study

[a] Lack of data

Table S6. Ratios of OCs for source identification in wild tilapia.

	(p,p'-DDE+p,p'-DDD)/p,p'-DDT	o,p'-DDT/p,p'-DDT	α-Endosulfan/β-Endosulfan	Light PCBs [a]	Medium PCBs [b]	Heavy PCBs [c]		(p,p'-DDE+p,p'-DDD)/p,p'-DDT	o,p'-DDT/p,p'-DDT	α-Endosulfan/β-Endosulfan	Light PCBs [a]	Medium PCBs [b]	Heavy PCBs [c]
	0.20	0.00	0.90	8.4%	7.8%	83.8%		1.41	0.14	0.09	19.9%	15.8%	64.3%
	0.13	0.01	1.17	8.8%	9.6%	81.7%		0.85	0.13	0.38	21.9%	18.3%	59.8%
	0.21	0.00	1.80	4.1%	8.5%	87.4%		2.57	0.14	0.26	16.6%	23.4%	59.9%
	0.22	0.00	1.18	7.9%	6.2%	85.9%		1.26	0.13	1.52	19.6%	21.1%	59.3%
Tiandong	0.11	0.00	0.96	7.9%	6.4%	85.7%	GG	1.10	0.10	0.17	26.2%	21.0%	52.8%
(TD)	0.19	0.00	0.53	5.2%	6.3%	88.5%		7.94	0.27	0.35	13.0%	18.3%	68.7%
	0.18	0.00	1.22	8.6%	8.5%	82.9%		6.60	0.17	0.23	17.4%	15.9%	66.7%
	0.17	0.00	3.16	6.1%	5.8%	88.1%		6.61	0.30	0.11	10.5%	10.4%	79.1%
	0.27	0.00	2.58	6.4%	6.0%	87.6%		11.8	0.41	1.20	18.9%	17.2%	64.0%
	0.22	0.00	4.84	5.9%	8.6%	85.5%		4.38	0.07	1.12	10.2%	11.1%	78.7%
	16.8	0.20	0.42	5.3%	9.5%	85.1%		3.03	0.00	0.42	5.7%	9.8%	84.6%
LA	11.5	0.15	0.19	9.4%	6.9%	83.6%	WX	3.73	0.48	0.79	4.2%	9.0%	86.8%
	7.69	0.31	0.56	13.3%	16.2%	70.4%		3.64	0.30	1.87	6.5%	4.3%	89.2%
	3.20	0.03	0.51	8.7%	11.0%	80.2%		5.38	0.43	0.23	8.7%	8.7%	82.7%
	4.22	0.04	0.22	5.0%	8.1%	86.8%		5.53	0.37	0.58	10.4%	11.3%	78.3%
	9.60	0.13	1.43	6.1%	11.7%	82.2%		6.81	0.20	0.81	12.7%	11.1%	76.2%
CZ	8.07	0.18	0.22	6.4%	10.1%	83.5%	PN	16.0	0.12	0.26	3.9%	13.9%	82.3%
	16.8	0.29	0.30	6.1%	8.5%	85.4%		4.71	0.09	1.03	9.4%	11.1%	79.6%
	5.30	0.03	0.28	4.3%	7.9%	87.7%		13.2	0.59	0.84	11.3%	18.4%	70.3%
	10.5	0.14	0.13	6.2%	8.8%	85.0%		7.99	0.19	0.23	6.3%	8.6%	85.1%

CZ	59.1	1.71	0.33	6.5%	7.5%	85.9%		21.7	0.24	0.46	8.6%	10.6%	80.8%
	11.5	0.51	4.05	5.8%	12.7%	81.5%	PN	44.9	0.49	1.21	8.1%	14.2%	77.7%
	12.9	0.00	0.35	7.9%	14.0%	78.1%		45.0	0.58	2.03	12.3%	17.3%	70.4%
	10.7	0.36	12.10	5.3%	10.0%	84.8%		11.6	0.31	0.31	10.6%	9.6%	79.8%
FS	10.8	0.00	0.25	8.4%	7.0%	84.6%		146	0.82	0.19	4.3%	10.4%	85.3%
	0.00	0.00	4.63	2.8%	3.4%	93.8%		94.1	0.00	0.46	7.5%	18.1%	74.4%
	9.00	0.32	0.09	12.2%	8.3%	79.4%		0.00	0.00	1.08	7.8%	9.8%	82.4%
	19.6	1.80	0.74	8.1%	8.3%	83.6%		0.00	0.00	2.03	5.0%	13.4%	81.6%
	9.16	0.00	0.34	5.3%	12.5%	82.2%	TX	0.00	0.00	0.41	3.3%	16.2%	80.5%
	6.92	0.00	1.58	5.5%	10.4%	84.1%		0.00	0.00	0.00	5.5%	14.3%	80.2%
	0.00	0.00	1.29	6.7%	11.6%	81.7%		0.00	0.00	1.99	6.2%	11.4%	82.4%
	5.82	0.17	0.11	3.8%	10.0%	86.2%		0.00	0.00	0.19	5.9%	11.2%	82.9%
NN	6.48	0.08	0.19	4.9%	9.9%	85.2%		0.00	0.00	1.12	5.6%	11.1%	83.4%
	7.48	0.39	0.62	5.1%	9.7%	85.2%		30.8	0.34	2.29	7.7%	13.3%	79.0%
	7.12	0.25	0.21	3.8%	7.3%	89.0%		14.5	0.36	0.34	9.0%	21.4%	69.6%
	9.83	0.12	0.22	4.3%	7.3%	88.4%	WZ	10.4	0.00	0.10	15.1%	16.3%	68.6%
	17.4	0.05	0.16	3.4%	7.1%	89.5%		17.9	0.00	0.25	11.7%	20.1%	68.2%
								0.00	0.00	0.54	15.4%	24.7%	59.9%

[a] Light PCBs with 2–3 chlorines;

[b] Medium PCBs with 4–6 chlorines;

[c] Heavy PCBs with 7–10 chlorines

Table S7. Correlation coefficient matrix for OC compounds (based on wet weight) and growth parameters of fish samples (n = 75).

	HCHs	DDTs	CHLs	Endosulfan	Drins	HCB	OCPs	PCBs	T-length[a]	B-mass[b]	Age	Lipid
HCHs	1.00											
DDTs	**0.339****[c]	1.00										
CHLs	-0.09	0.14	1.00									
E-sulfan	-0.18	-0.14	**0.317****	1.00								
Drins	0.02	0.00	-0.05	-0.16	1.00							
HCB	**0.830****	**0.326****	-0.08	**-0.258***	0.10	1.00						
OCPs	-0.11	**0.362****	**0.411****	**0.466****	-0.11	-0.07	1.00					
PCBs	0.11	**0.491****	0.22	-0.15	-0.06	0.14	**0.545****	1.00				
T-length	0.11	**0.350****	**0.473****	-0.17	-0.13	0.11	**0.534****	**0.492****	1.00			
B-mass	0.03	**0.351****	**0.530****	-0.12	-0.16	0.07	**0.632****	**0.444****	**0.910****	1.00		
Age	0.20	**0.266***	**0.398****	-0.03	-0.11	0.08	**0.387****	**0.305****	**0.485****	**0.529****	1.00	
Lipid	**0.54****	**0.80****	-0.07	-0.16	-0.06	**0.47****	0.14	**0.402****	0.23	0.14	**0.277***	1.00

[a]: Total length; [b]: Body mass; [c]: Significance level: ** $p < 0.01$； * $p < 0.05$ indicated that the difference was significant and the number was in bold.

Table S8. Correlation coefficient matrix for OC compounds and growth parameters of fish samples (n = 75). Correlation coefficient matrix for individual OCs and growth parameters of fish samples (n = 75).

	α-HCH	β-HCH	γ-HCH	δ-HCH	o,p'-DDE	p,p'-DDE	o,p'-DDD	p,p'-DDD	o,p'-DDT	o,p'-DDT	TC	CC	Endosulfan sulfate	Light PCB	Medium PCB	Heavy PCB
α-HCH	1.000															
β-HCH	**0.719****	1.000														
γ-HCH	**0.723****	**0.768****	1.000													
δ-HCH	**0.514****	0.190	**0.454****	1.000												
o,p'-DDE	**0.318****	**0.696****	**0.480****	0.081	1.000											
p,p'-DDE	**0.250***	**0.674****	**0.471****	0.041	**0.901****	1.000										
o,p'-DDD	0.200	**0.704****	**0.417****	0.037	**0.889****	**0.905****	1.000									
p,p'-DDD	0.225	**0.701****	**0.433****	0.048	**0.863****	**0.944****	**0.967****	1.000								
o,p'-DDT	0.133	**0.611****	**0.347****	0.025	**0.845****	**0.893****	**0.970****	**0.973****	1.000							
o,p'-DDT	0.122	**0.586****	**0.343****	0.029	**0.803****	**0.894****	**0.946****	**0.976****	**0.988****	1.000						
TC	0.021	0.004	-0.018	-0.012	-0.001	-0.047	-0.008	-0.022	-0.010	-0.020	1.000					
CC	-0.089	-0.105	-0.099	-0.014	-0.152	-0.161	-0.087	-0.090	-0.062	-0.019	-0.040	1.000				
Endosulfan sulfate	-0.164	-0.198	-0.211	-0.083	-0.202	-0.219	-0.131	-0.141	-0.101	-0.047	-0.079	**0.843****	1.000			
Light PCB	**0.431****	**0.826****	**0.587****	0.140	**0.826****	**0.871****	**0.865****	**0.892****	**0.813****	**0.820****	0.069	-0.042	-0.074	1.000		
Medium PCB	**0.460****	**0.776****	**0.655****	0.227	**0.782****	**0.837****	**0.766****	**0.768****	**0.676****	**0.671****	0.064	-0.159	**-0.231***	**0.851****	1.000	
Heavy PCB	0.014	0.108	0.061	-0.177	0.182	0.160	0.147	0.161	0.139	0.147	-0.105	0.190	0.219	0.190	0.109	1.000
Total length (cm)	0.086	0.105	0.066	0.175	0.080	0.105	0.110	0.146	0.116	0.117	-0.047	-0.105	-0.110	0.139	0.035	0.011
Body mass (g)	0.018	0.031	0.011	0.122	0.037	0.065	0.053	0.098	0.074	0.078	-0.076	-0.093	-0.109	0.055	-0.021	-0.025
Age (year)	0.193	0.217	0.172	0.185	0.140	0.183	0.155	0.188	0.138	0.149	0.014	-0.031	-0.030	**0.247***	0.162	0.112
Lipid (%)	**0.624****	**0.370****	**0.471****	**0.600****	0.091	0.031	0.048	0.044	0.011	0.009	0.027	-0.026	-0.093	0.210	0.213	0.027

Significance level: **$p < 0.01$； * $p < 0.05$ indicated that the difference was significant and the number was in bold.

Table S9. Estimated daily intakes (EDIs) of OCPs and PCBs in fish by humans using maximum concentrations of OCPs and PCBs (ng/g, ww) in collected wild tilapia.

		ADIs[a]	Sample sites									
			WX	FS	LA	TD	CZ	GG	PN	WZ	TX	NN
HCHs	Concentration	5000	0.01	0.01	0.01	0.01	0.01	0.1	0.05	0.08	0.02	0.01
	EDI[b](ng/kg/day)		0.01	0.01	0.01	0.01	0.02	0.1	0.05	0.08	0.02	0.01
DDTs	Concentration	10000	0.11	0.17	0.17	0.12	0.54	6.93	0.89	0.21	0.42	0.23
	EDI (ng/kg/day)		0.12	0.18	0.18	0.13	0.56	7.19	0.92	0.22	0.43	0.24
CHLs	Concentration	500	0.02	0.03	0.08	0.12	0.03	0.04	0.02	0.03	0.02	0.03
	EDI (ng/kg/day)		0.02	0.03	0.08	0.13	0.03	0.04	0.03	0.03	0.02	0.03
Endosulfan	Concentration		0.07	0.29	0.15	11.56	0.22	0.68	0.37	0.29	0.06	0.18
	EDI (ng/kg/day)		0.08	0.3	0.16	11.99	0.23	0.7	0.39	0.3	0.06	0.18
Drins	Concentration	100	0.09	0.1	0.1	0.07	0.11	0.14	0.12	0.1	0.07	0.1
	EDI (ng/kg/day)		0.09	0.11	0.1	0.08	0.11	0.15	0.12	0.11	0.07	0.1
HCB	Concentration	600	0.03	0.04	0.02	0.02	0.05	0.11	0.09	0.08	0.03	0.05
	EDI (ng/kg/day)		0.03	0.04	0.02	0.02	0.06	0.11	0.09	0.08	0.03	0.05
PCBs	Concentratio		0.11	0.12	0.09	0.12	0.11	0.18	0.13	0.1	0.12	0.1
	EDI (ng/kg/day)		0.12	0.13	0.09	0.12	0.12	0.19	0.14	0.11	0.13	0.11
OCP	Concentration		0.29	0.49	0.43	11.85	0.93	7.47	1.14	0.73	0.53	0.48
	EDI (ng/kg/day)		0.3	0.51	0.45	12.29	0.97	7.75	1.19	0.75	0.55	0.5

[a] Acceptable daily intakes (ng/kg/d) (WHO, 2009)

[b] Calculated by the method shown in Test LA.

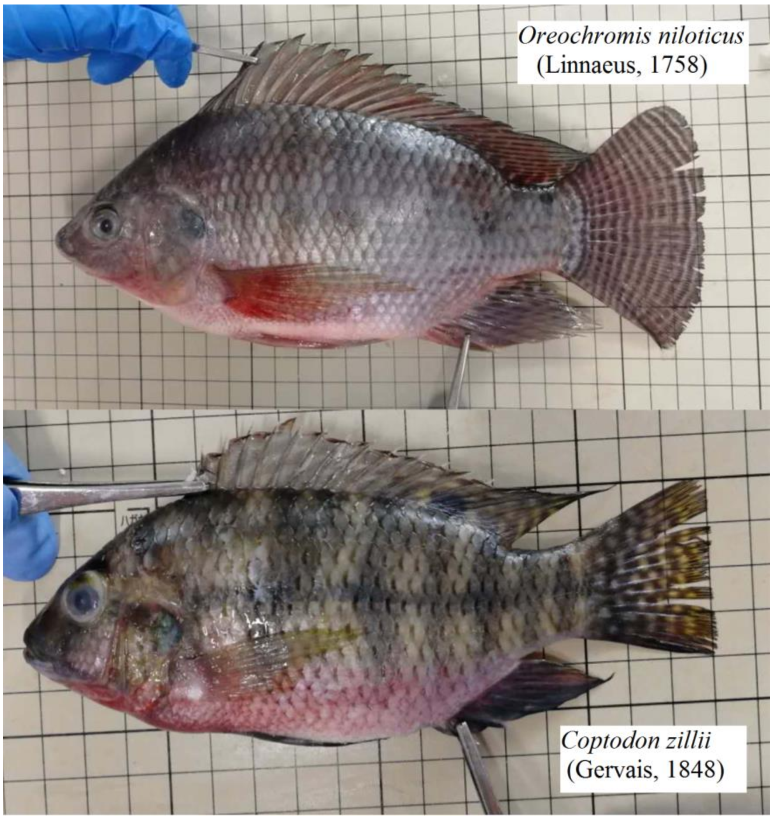

Fig. S1. Studied wild tilapias were purchased from the main river in Guangxi, South China.

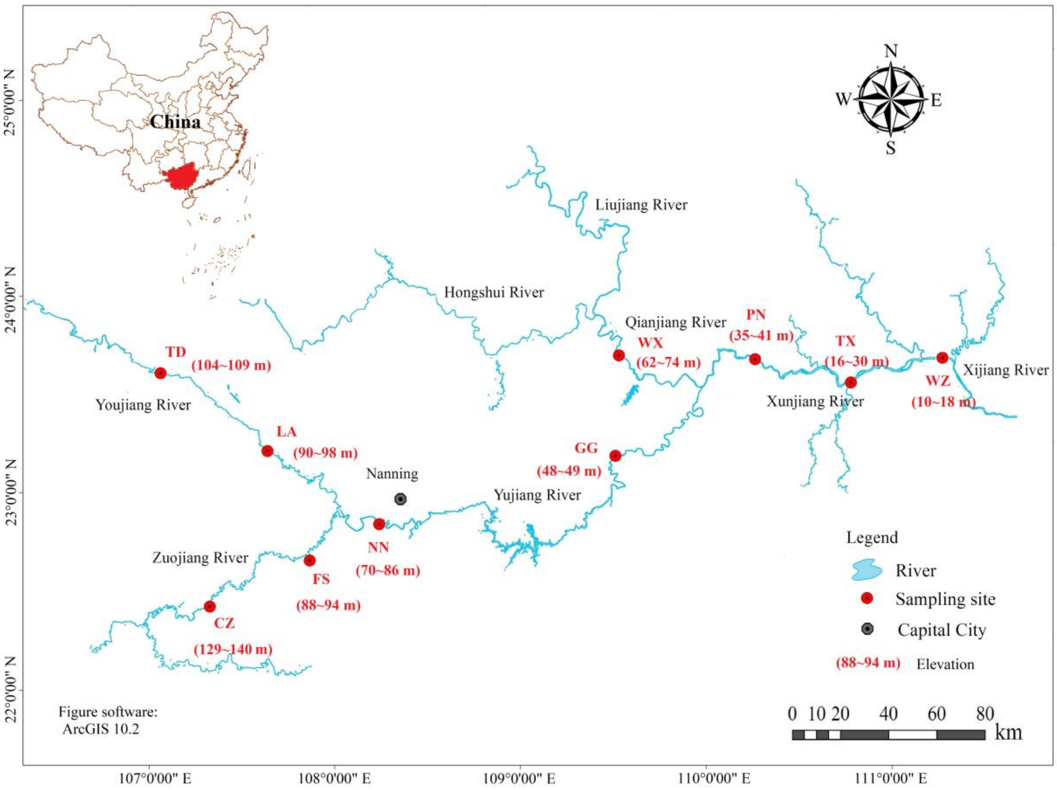

Fig. S2. Distribution map of sampling locations in the main river in Guangxi, South China. TD: Tiandong County; LA: Longan County; CZ: Chongzuo City; FS: Fusui City; NN: Nanning City; GG: Guigang City; WX: Wuxuan County; PN: Pingnan City; TX: Tengxian County; WZ: Wuzhou City.

References

Adu-Kumi, S., Kawano, M., Shiki, Y., Yeboah, P.O., Carboo, D., Pwamang, J., Morita, M., Suzuki, N. Organochlorine pesticides (OCPs), dioxin-like polychlorinated biphenyls (dl-PCBs), polychlorinated dibenzo-p-dioxins and polychlorinated dibenzo furans (PCDD/Fs) in edible fish from Lake Volta, Lake Bosumtwi and Weija Lake in Ghana. Chemosphere 81 (2010), 675-684.

Botaro, D., Torres, J.P.M., Malm, O., Rebelo, M.F., Henkelmann, B., Schramm, K. Organochlorine pesticides residues in feed and muscle of farmed Nile tilapia from Brazilian fish farms. Food Chem. Toxicol. 49 (2011), 2125-2130.

Covaci, A., Gheorghe, A., Hulea, O., Schepens, P. Levels and distribution of organochlorine pesticides, polychlorinated biphenyls and polybrominated diphenyl ethers in sediments and biota from the Danube Delta, Romania. Environ. Pollut. 140 (2006), 136-149.

Ding, Y., Wu, Z.Q., Zhang, R.J., Yu, K.F., Wang, Y.H., Zou, Q., Zeng, W.B., Han, M.W. Organochlorines in fish from the coastal coral reefs of Weizhou Island, South China Sea: levels, sources, and bioaccumulation. Chemosphere 232 (2019), 1-8.

Dougherty, C.P., Holtz, S.H., Reinert, J.C., Panyacosit, L., Axelrad, D.A., Woodruff, T.J. Dietary Exposures to Food Contaminants across the United States. Environ. Res. 84 (2000), 170-185.

Guangxi Statistics Bureau. Guangxi Statistical Yearbook in 2018. China Statistics Press., Beijing. 2019.

Guo, J.Y., Wu, F.C., Shen, R.L., Zeng, E.Y. Dietary intake and potential health risk of DDTs and PBDEs via seafood consumption in South China. Ecotox. Environ. Safe. 73 (2010), 1812-1819.

Guo, L.L., Qiu, Y.W., Zhang, G., Zheng, G.J., Lam, P.K.S., Li, X.D. Levels and bioaccumulation of organochlorine pesticides (OCPs) and polybrominated diphenyl ethers (PBDEs) in fishes from the Pearl River estuary and Daya Bay, South China. Environ. Pollut. 152 (2008), 604-611.

Jiang, Q.T., Lee, T.K.M., Chen, K., Wong, H.L., Zheng, J.S., Giesy, J.P., Lo, K.K.W., Yamashita, N., Lam, P.K.S. Human health risk assessment of organochlorines associated with fish consumption in a coastal city in China. Environ. Pollut. 136 (2005), 155-165.

Kong, K., Cheung, K., Wong, C., Wong, M. Residues of DDTs, PAHs and some heavy metals in fish (Tilapia) collected from Hong Kong and mainland China. J. Environ. Sci. Health. A. Tox. Hazard. Subst. Environ. Eng. 40 (2005), 2105-2115.

Mai, B.X., Zeng, E.Y., Luo, X.J., Yang, Q.S., Zhang, G., Li, X.D., Sheng, G.Y., Fu, J.M. Abundances, depositional fluxes, and homologue patterns of polychlorinated biphenyls in dated sediment cores from the Pearl River Delta, China. Environ. Sci. Technol. 39 (2005), 49-56.

Manirakiza, P., Covaci, A., Nizigiymana, L., Ntakimazi, G., Schepens, P. Persistent chlorinated pesticides and polychlorinated biphenyls in selected fish species from Lake Tanganyika, Burundi, Africa. Environ. Pollut. 117 (2002), 447-455.

Ogwok, P., Muyonga, J.H., Sserunjogi, M.L. Pesticide Residues and Heavy Metals in Lake Victoria Nile Perch, Lates niloticus, Belly Flap Oil. B. Environ. Contam. Tox. 82 (2009), 529-533.

Polder, A., Müller, M.B., Lyche, J.L., Mdegela, R.H., Nonga, H.E., Mabiki, F.P., Mbise, T.J., Skaare, J.U., Sandvik, M., Skjerve, E., Lie, E. Levels and patterns of persistent organic pollutants (POPs) in tilapia (Oreochromis sp.) from four different lakes in Tanzania: Geographical differences and implications for human health. Sci. Total Environ. 488-489 (2014), 252-260.

Qiao, M., An, T.C., Zeng, X.Y., Zhang, D.L., Li, G.Y., Zhang, G.X., Guo, J. Levels and sources of

typical persistent organic pollutants in drinking water sources of Xijiang River in Guangdong province. Ecology and Environmental Sciences. 19 (2010), 556-561.

Said, T.O., El Moselhy, K.M., Rashad, A.A.M., Shreadah, M.A. Organochlorine Contaminants in Water, Sediment and Fish of Lake Burullus, Egyptian Mediterranean Sea. B. Environ. Contam. Tox. 81 (2008), 136-146.

Sapozhnikova, Y., Bawardi, O., Schlenk, D. Pesticides and PCBs in sediments and fish from the Salton Sea, California, USA. Chemosphere 55 (2004), 797-809.

Sun, Y.X., Zhang, Z.W., Xu, X.R., Hu, Y.X., Luo, X.J., Cai, M.G., Mai, B.X. Bioaccumulation and biomagnification of halogenated organic pollutants in mangrove biota from the Pearl River Estuary, South China. Mar. Pollut. Bull. 99 (2015), 150-156.

USEPA. Guidance for assessing chemical contaminant, data for use in fish advisories. Fish sampling and analysis, 3rd ed. Office of Water, Washington, DC, 2000.

FAO/WHO. Pesticide Residues in Food. Joint FAO/WHO Meeting on Pesticide Residues Report of the Jointmeeting of the FAO Panel of Experts on Pesticide Residues in Food and the Environment and the WHO Core Assessment Group on Pesticide Residues. World Health Organization, Food and Agriculture Organization of the United Nations, Rome, 2009.
